# Phenotypic and Genotypic Characteristics of *Staphylococcus aureus* Nasal Strains Isolated from Students of the Pomeranian Medical University in Szczecin, Poland: A Cross-Sectional Study

**DOI:** 10.3390/toxins18050237

**Published:** 2026-05-21

**Authors:** Paweł Kwiatkowski, Helena Masiuk, Agata Pruss, Stefania Giedrys-Kalemba, Piotr Baszuk, Iwona Wojciechowska-Koszko, Monika Sienkiewicz

**Affiliations:** 1 Department of Diagnostic Immunology, Pomeranian Medical University in Szczecin, Powstancow Wielkopolskich 72, 70-111 Szczecin, Poland; iwona.wojciechowska.koszko@pum.edu.pl; 2 Department of Medical Microbiology, Pomeranian Medical University in Szczecin, Powstancow Wielkopolskich 72, 70-111 Szczecin, Poland; helena.masiuk@pum.edu.pl (H.M.); kalemba@mp.pl (S.G.-K.); 3 Department of Laboratory Medicine, Pomeranian Medical University in Szczecin, Powstancow Wielkopolskich 72, 70-111 Szczecin, Poland; agata.pruss@pum.edu.pl; 4 Department of Genetics and Pathology, International Hereditary Cancer Center, Pomeranian Medical University in Szczecin, Unii Lubelskiej 1, 71-252 Szczecin, Poland; piotr.baszuk@pum.edu.pl; 5 Department of Pharmaceutical Microbiology and Microbiological Diagnostics, Medical University of Lodz, 90-151 Lodz, Poland

**Keywords:** nasal carriage, students, antimicrobial susceptibility testing, virulence factors, PFGE, *Staphylococcus aureus*

## Abstract

*Staphylococcus aureus* nasal carriage contributes to asymptomatic transmission in both community and healthcare settings. This study aimed to characterize *S. aureus* strains isolated from students of the Pomeranian Medical University in Szczecin, Poland, using phenotypic and genotypic methods. A total of 175 *S. aureus* strains were isolated from the nasal vestibules of 800 students between 2014 and 2015. Species identification and antimicrobial susceptibility testing were performed using standard microbiological methods, while virulence-associated genes and *agr* groups were analyzed using Single-PCR and Multiplex-PCR assays. Genotypic diversity was assessed by pulsed-field gel electrophoresis (PFGE). The prevalence of *S. aureus* nasal carriage among students was 21.9% and did not differ according to faculty or year of study. Most isolates (84.0%) were susceptible to all tested antibiotics, and no methicillin-resistant *S. aureus* (MRSA) strains were detected. All strains carried the *hla* gene, whereas *hld* and *hlg* were identified in 93.7% and 93.1% of isolates, respectively. In addition, the *tst* gene was detected in 22.3% of strains, while the *lukS-PV*/*lukF-PV* genes were identified in only one isolate (0.6%). The most prevalent enterotoxin genes were *sep* (17.1%) and *sea* (13.7%), whereas genes of the *egc* cluster, including *seg*, *sei*, and *seo*, were detected in 53.7% of isolates. Significant associations were observed between specific *egc* gene combinations and superantigen gene profiles, including increased frequencies of *sec*, *sel*, and *tst* genes (*p* < 0.001). The predominant *agr* type was *agr-1* (49.7%), followed by *agr-3* (28.6%) and *agr-2* (20.0%). Strains carrying *agr-1* more frequently harbored the *g i m n o* cluster as well as the *sec*, *sel*, and *sep* genes, whereas *agr-3*-positive isolates were significantly associated with the *g i m o u* and *g i o u* clusters and with the presence of *tst*, *sea*, and *seh* genes (*p* < 0.05). PFGE analysis demonstrated substantial genetic heterogeneity among the isolates, with no evidence of a predominant clonal lineage. These findings indicate a heterogeneous, non-epidemic population structure of *S. aureus* strains circulating among university students and highlight the considerable diversity and interrelationships of virulence-associated genetic profiles within this population.

## 1. Introduction

*Staphylococcus aureus* is a Gram-positive, facultatively anaerobic bacterium that belongs to the Staphylococcaceae family [1]. It often appears as clusters resembling grape bunches under the microscope and is characterized by its ability to produce coagulase and the golden pigment staphyloxanthin [2]. The clinical significance of *S. aureus* lies in its versatility, ability to persist in the human host, and growing resistance to antibiotics, particularly methicillin, which makes it a significant concern in both hospital and community settings [3,4].

The pathogenic potential of *S. aureus* is primarily associated with a broad arsenal of virulence factors. These include surface adhesins such as clumping factors, fibronectin-binding proteins, and collagen-binding adhesins, which facilitate bacterial attachment to host tissues and medical devices. Enzymes such as coagulase, hyaluronidase, lipases, nucleases, and proteases contribute to immune evasion, tissue degradation, and dissemination within the host [5,6]. *S. aureus* produces numerous exotoxins, some of which function as superantigens (SAgs) that excessively activate T cells and trigger a strong cytokine response. These include toxic shock syndrome toxin-1 (TSST-1) and staphylococcal enterotoxins (SEs), which are implicated in conditions such as toxic shock syndrome and food poisoning, and exfoliative toxins (ETs), which contribute to skin disorders such as staphylococcal scalded skin syndrome and bullous impetigo [7]. Another notable virulence factor is Panton-Valentine leukocidin (PVL), a cytotoxin associated with necrotizing infections, which promotes tissue damage by destroying leukocytes and intensifying inflammation [8].

*S. aureus* is also commonly a commensal organism, with nasal carriage rates in the general population estimated between 20 and 30% [9]. This asymptomatic carrier state serves as a critical reservoir for transmission and increases the risk of endogenous infections, especially among immunocompromised individuals and patients undergoing surgery [10,11]. Certain groups, such as healthcare workers, patients with chronic illnesses, and individuals exposed to the hospital environment, demonstrate a higher prevalence of colonization. Key risk factors include host factors (e.g., compromised immunity, atopic dermatitis), prior antibiotic use, and environmental exposure to colonized individuals or contaminated surfaces [12].

Nasal carriers are considered a significant reservoir for *S. aureus*, increasing the risk of endogenous infections, particularly surgical site and bloodstream infections, as well as pneumonia and osteomyelitis [13]. Nasal carriage of *S. aureus* in healthy individuals is a key factor contributing to the spread of staphylococcal infections [14]. Its prevalence among outpatients typically ranges from 20% to 40%, with significant geographic variation [15]. The highest rates have been reported in South America (57.8%) and the lowest in Indonesia (9.1%). In Europe, approximately 21.6% of residents are nasal carriers of *S. aureus* [16]. A multicenter study involving over 32,000 patients from nine European countries showed national prevalence rates ranging from 14.1% in Hungary to 29.8% in Sweden. In Poland, the carriage rate is comparable to the European average, reaching nearly 20% in healthy individuals [17]. These epidemiological data emphasize the need for continuous monitoring of *S. aureus* carriage, particularly in populations with repeated healthcare exposure, where transmission dynamics may differ from the general population.

Students of medical universities, particularly those involved in clinical internships and hospital rotations, constitute a unique population potentially exposed to hospital-acquired strains of *S. aureus*, including MRSA. Close contact with healthcare personnel, patients, and contaminated environments facilitates the acquisition and transmission of these strains. Students who become colonized during clinical practice may unknowingly act as vectors, contributing to the dissemination of *S. aureus* between hospital wards or institutions [18,19]. MRSA remains one of the most clinically significant multidrug-resistant pathogens associated with both healthcare- and community-acquired infections. However, increasing attention has also been directed toward methicillin-susceptible *S. aureus* (MSSA) strains, which may harbour resistance mechanisms against other clinically important antimicrobial agents, including macrolide-lincosamide-streptogramin B (MLS_B_) antibiotics, fluoroquinolones such as ciprofloxacin (CIP), and mupirocin (MUP) used for decolonization therapy. Resistance to these agents is commonly associated with the presence of *erm* genes, mutations affecting DNA gyrase and topoisomerase IV, and *mupA*/*mupB* genes, respectively, which may contribute to therapeutic limitations and facilitate persistence and transmission of colonizing strains [20].

Despite increasing global data on *S. aureus* carriage, there remains a limited understanding of the molecular epidemiology and virulence gene distribution among medical students, particularly in Central and Eastern Europe. In addition, few studies have simultaneously evaluated phenotypic resistance patterns, virulence determinants, and clonal diversity within a single student population exposed to early clinical training [21,22,23]. The Pomeranian Medical University (PMU) cohort is of particular interest due to its structured early clinical exposure across multiple healthcare disciplines, which may influence colonization dynamics and strain diversity differently compared to non-medical or later-stage healthcare populations.

Given the epidemiological relevance of *S. aureus* colonization among future healthcare professionals, this cross-sectional study aimed to perform phenotypic and genotypic analyses of *S. aureus* strains isolated from students across various faculties and years at the PMU in Szczecin, Poland. Hence, the primary goal was to determine the prevalence of nasal carriage in this population. Additionally, the study assessed the antibiotic susceptibility of the *S. aureus* strains, the presence of genes encoding selected virulence factors, and the genetic relatedness of the strains to better understand the potential sources and transmission dynamics of colonizing strains.

## 2. Results

### 2.1. Prevalence of Nasal S. aureus Carriage Among Students

Of nasal vestibule swabs collected from 800 students, 175 (21.9%) tested positive for *S. aureus*. However, there was no statistically significant difference in the proportion of carriers and non-carriers in the study population (chi-squared test; *p* = 0.152). The percentage of positive cultures ranged from 9.1% to 26.9% across different fields of study. The highest rate was observed among 5th-year medical analytics students, while the lowest was among 2nd-year medical biotechnology students. Detailed results are presented in Table 1.

To confirm the identity of the *S. aureus* strains, phenotypic and molecular tests were performed. All strains were confirmed as *S. aureus* based on the presence of protein A, detection of the *nuc* gene fragment, and the production of both free and bound coagulase (clumping factor).

Concerning sex, no significant association was observed with *S. aureus* carriage. Among the 175 carriers identified in the study, 52 (29.7%) were male and 123 (70.3%) were female (Table 2). Statistical analysis revealed no significant difference in carriage rates between male and female students (chi-squared test; *p* = 0.567), indicating that gender did not influence colonization likelihood in the studied population. Additionally, no statistically significant association between gender and *S. aureus* carriage was observed within individual fields of study and years of study (Fisher’s exact test; *p* = 0.583).

### 2.2. AST Results for S. aureus Strains

The majority of *S. aureus* strains (*n* = 147; 84.0%) were susceptible to all tested antimicrobial agents (Table 3). No strains were resistant to cefoxitin (FOX), indicating the absence of MRSA strains in the studied group. Resistance to the remaining antibacterial agents among the *S. aureus* strains was as follows: MUP (*n* = 3; 1.7%), trimethoprim/sulfamethoxazole (SXT; *n* = 1; 0.6%), CIP (*n* = 1; 0.6%), erythromycin (E; *n* = 24; 13.7%), and clindamycin (CC; *n* = 24; 13.7%).

Most of the E- and CC-resistant strains exhibited an inducible macrolide-lincosamide-streptogramin B resistance phenotype (iMLS_B_; *n* = 22; 91.7%), while the remaining two showed a constitutive macrolide-lincosamide-streptogramin B resistance phenotype (cMLS_B_). No macrolide-lincosamide (MS_B_) phenotype was detected.

Among the 28 (16.0%) resistant strains, six antimicrobial resistance patterns were identified. The most frequently observed resistance pattern was iMLS_B_, found in 21 (12.0%) strains. The remaining phenotypes were represented by one or two strains each. Detailed data are listed in Table 3.

The proportion of drug-resistant *S. aureus* strains isolated from students varied across different fields of study. No resistant strains were identified among 3rd-year medical analytics, 1st-year dietetics, or 2nd-year midwifery students. The highest proportions were observed among strains from 2nd-year medical biotechnology students (2 out of 3; 66.7%) and 1st-year midwifery students (4 out of 6; 66.7%), with the iMLS_B_ resistance phenotype being predominant. In study programs with a larger number of strains (>20), such as medicine and dentistry, the proportion of resistant strains ranged from 15.8% to 20.0%. Detailed results are presented in Table 3.

### 2.3. Molecular Analysis of S. aureus Strains

#### 2.3.1. Prevalence of Genes Encoding Hemolysins, TSST-1, ETs, and Selected SEs

Molecular analyses revealed that all tested *S. aureus* strains carried the *hla* gene. The *hld* and *hlg* genes were detected in 93.7% and 93.1% of strains, respectively. The *hld* gene was found in 41.7% of strains from 2nd-year paramedic students, 89.5% from 3rd-year medical students, and 100% from all other study fields. The *hlg* gene was detected in nearly all strains from most fields, although slightly lower frequencies (73.3–92.1%) were observed among strains from 1st-year nursing, 1st-year midwifery, and 2nd- and 3rd-year medical students. In contrast, the *hlb* gene was found in only 9 out of 175 strains (5.1%), including 1 from a 5th-year medical analytics student, 2 from 3rd-year medical and 2nd-year paramedic students, and 4 from 2nd-year dentistry students.

Among the virulence genes associated with toxin production, *tst* was detected in 39 *S. aureus* strains (22.3%). Its prevalence varied across fields and years of study, ranging from 6.7% to 66.7%; notably, it was absent in all strains from 1st-year midwifery students. The *lukS-PV*/*lukF-PV* gene was identified in only one isolate (0.6%) from a 2nd-year paramedic student.

The *eta* and *etd* were detected in 1.1% and 1.7% of strains, respectively. The *eta* gene was found in strains from a 2nd-year dentistry student and a 1st-year nursing student. The *etd* gene was identified in three strains originating from 1st-year dietetics students and 2nd- and 3rd-year medical students. The *etb* gene was not detected in any of the tested strains.

The analysis of SE genes prevalence among *S. aureus* strains collected from students across different study fields and years revealed marked variability among groups. The most frequently detected SE gene was *sep* (17.1%), followed by *sea* (13.7%), *sec* (7.4%), and *sel* (7.4%). Notably, the *sel* gene co-occurred with *sec* in 92.3% of *sec*-positive strains (*p* < 0.001), confirming a strong genetic linkage between these enterotoxin genes. Other genes, including *seb*, *sed*, *seh*, *sej*, *sek*, *seq*, and *ser*, were rarely identified (ranging from 0.6% to 2.3%), while *see* was not detected in any isolate. A higher diversity and frequency of SE genes were observed among strains from students of medical analytics (3rd and 5th year), medicine (2nd and 3rd year), and dentistry (2nd year). For instance, the *sea* gene was found in 62.5% of *S. aureus* strains from 3rd-year medical analytics students, while *sec* and *sel* were present in 42.9% of strains from 5th-year students of the same program. In contrast, *S. aureus* strains from students of midwifery (1st and 2nd year), nursing (1st year), dietetics (1st year), and paramedic science (2nd year) showed lower SE gene diversity and lower SE gene frequency, with most SE genes detected sporadically or not at all.

A summary of the frequencies of genes encoding hemolysins, TSST-1, ETs, and selected SEs is provided in Table 4.

#### 2.3.2. Prevalence of the *egc* Cluster Genes

The analyzed *S. aureus* strains most frequently carried *seg*–94 (53.7%), *sei*–94 (53.7%), *seo*–94 (53.7%), *sem*–78 (44.6%), *seu*–52 (29.7%), and *sen*–45 (25.7%) genes. A total of 81 strains (46.3%) lacked any *egc* cluster genes. The *egc* cluster genes occurred in five combinations: *g i m n o u* (*n* = 4; 2.3%), *g i m n o* (*n* = 41; 23.4%), *g i m o u* (*n* = 32; 18.3%), *g i m o* (*n* = 1; 0.6%), and *g i o u* (*n* = 16; 9.1%). The most prevalent strains carried the following gene combinations: *g i m n o* (23.4%) and *g i m o u* (18.3%).

The *egc* cluster genes were detected in *S. aureus* strains isolated from students of different study fields and years in various combinations (1, 2, or 3) and at different frequencies (2.5–71.4%). Among *egc*-positive strains, the dominant gene combination *g i m n o* was most commonly found in fifth-year medical analytics students—five of seven strains (71.4%), whereas the *g i m o u* genes were identified in two of three strains (66.7%) isolated from second-year medical biotechnology students. Similarly, the absence of the *egc* operon genes was observed in 14.3–60.0% of strains, depending on the study field and year from which *S. aureus* was isolated. The frequency of *egc* cluster gene combinations in *S. aureus* strains, by study field and year, is summarized in Table 5.

Additionally, Fisher’s exact test was used to assess the relationship between the occurrence of individual *egc* clusters and the presence of virulence genes, which revealed significant associations between specific gene combinations and SAgs profiles. In the *g i m n o* cluster, the *sec* (OR = 53.43; 95% CI = 7.39–2337.16; *p* < 0.001), *sed* (OR is not calculable; 95% CI is not calculable; *p* = 0.012), *sej* (OR is not calculable; 95% CI is not calculable; *p* = 0.012), *sel* (OR = 23.6; 95% CI = 4.79–229.17; *p* < 0.001), and *ser* (OR is not calculable; 95% CI is not calculable; *p* = 0.012) genes were detected significantly more frequently. A borderline association was observed for the *sea* gene within the *g i m o u* cluster (OR = 2.63; 95% CI = 0.87–7.43; *p* = 0.05). In contrast, in the *g i m o u* and *g i o u* clusters, a significantly higher occurrence of the *tst* gene was observed (OR = 15.89; 95% CI = 6.07–44.73; *p* < 0.001 and OR = 7.35; 95% CI = 2.22–26.74; *p* < 0.001, respectively).

The analysis also included the relationship between virulence profiles and the absence of the *egc* operon. In the non-*egc* group, only the *sep* gene occurred significantly more often (OR = 50.96; 95% CI = 7.98–2116.56; *p* < 0.001), accounting for 96.7% of all *sep*-positive strains.

#### 2.3.3. *agr* System Genes

The *agr* system genes were detected in all analyzed strains (Table 6). The most frequently identified was *agr-1* (*n* = 87; 49.7%). Lower frequencies were observed for *agr-3* (*n* = 50; 28.6%) and *agr-2* (*n* = 35; 20.0%), whereas *agr-4* was detected in only 3 (1.7%) strains.

The occurrence of *agr* system genes varied across the different study programs (Table 6). The most frequently detected gene, *agr-1* (87 strains), was isolated in all programs, ranging from 33.3% to 85.7% of strains. Similarly, *agr-3* (50 strains) was detected in all programs, but at lower frequencies (1.7–66.7%). *agr-2* (35 strains) was isolated in most programs (12.5–40.0%), except for fifth-year medical analytics and second-year medical biotechnology. Three strains carrying the *agr-4* gene were isolated in the following programs: third-year medicine, first-year nursing, and first-year midwifery.

In addition, the relationship between the *agr* genes and the presence of individual *egc* clusters was assessed using Fisher’s exact test. In isolates carrying the *agr-1* gene, the *g i m n o* cluster was detected significantly more frequently (OR = 6.05; 95% CI = 2.50–16.35; *p* < 0.001), whereas strains harboring the *agr-3* gene more often carried the *g i m o u* and *g i o u* clusters (*p* < 0.001—OR and 95% CI are not calculable in both cases). In contrast, the *g i m n o u* cluster occurred significantly more frequently among strains positive for the *agr-4* gene (OR is not calculable; 95% CI is not calculable; *p* < 0.001).

The analysis of associations between *agr* system and SAg genes showed that strains positive for the *agr-1* gene more frequently harbored the *sec* (OR is not calculable; 95% CI is not calculable; *p* < 0.001), *sel* (OR = 13.77; 95% CI = 1.95–600.02; *p* = 0.001), and *sep* (OR = 8.85; 95% CI = 2.86–36.68; *p* < 0.001) genes. In contrast, strains positive for the *agr-3* gene were more often positive for the *tst* (OR = 29.02; 95% CI = 10.67–90.38; *p* < 0.001), *sea* (OR = 2.95; 95% CI = 1.11–7.87; *p* = 0.026), and *seh* (OR is not calculable; 95% CI is not calculable; *p* = 0.006) genes.

#### 2.3.4. Genetic Relatedness of the *S. aureus* Strains

**Figure 1:**
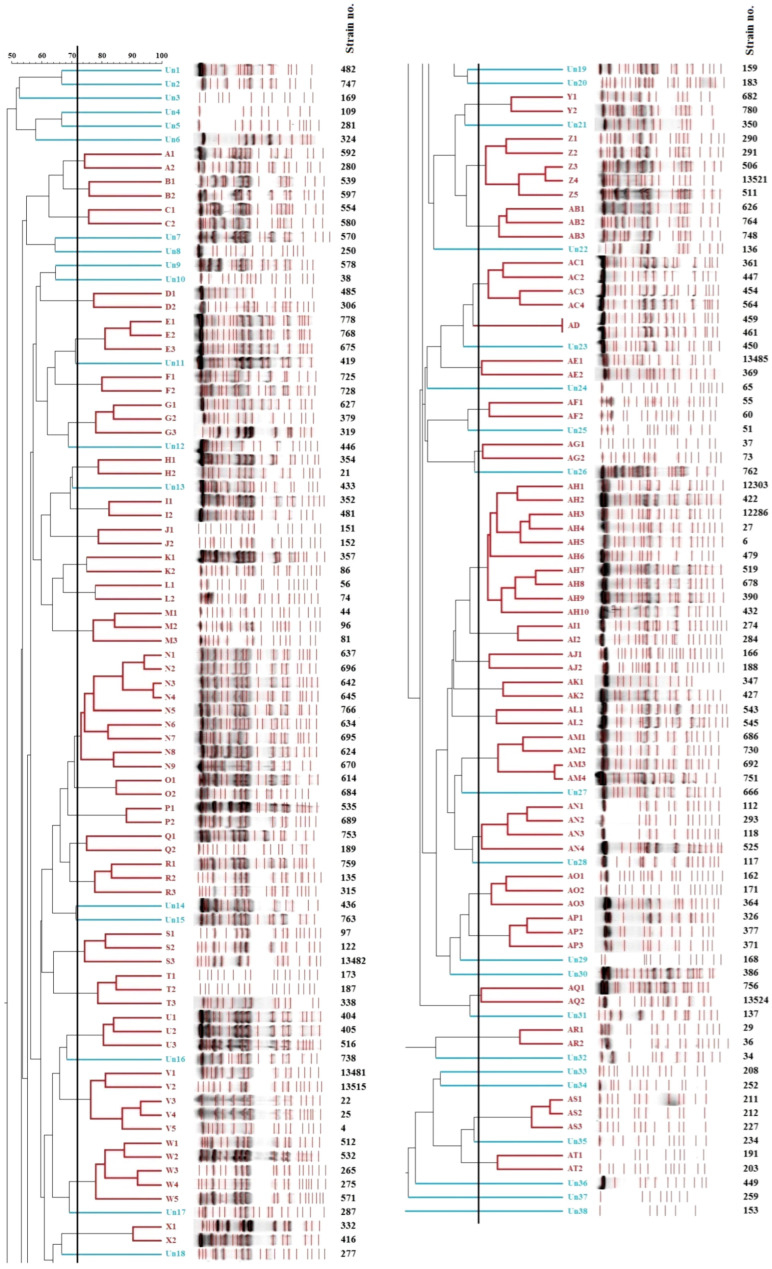
Dendrogram showing the genetic relatedness of *Staphylococcus aureus* strains obtained from the nasal vestibules of students (Sab = 72.2%; black line). Red labels indicate obtained genotypes, whereas blue labels indicate unique strains (Un). MA3—medical analytics, 3rd year; MA5—medical analytics, 5th year; MB2—medical biotechnology, 2nd year of first-cycle; D1—dietetics, 1st year; MED2—medicine, 2nd year; MED3—medicine, 3rd year; DENT2—dentistry, 2nd year; NUR1—nursing, 1st year; MID1—midwifery, 1st year; MID2—midwifery, 2nd year; PAR2—paramedic, 2nd year.

Genetic relatedness analysis of 175 *S. aureus* strains obtained from the nasal vestibules of students was performed using the PFGE method and analyzed with FPQuest software. Among the 175 analyzed strains, 169 yielded interpretable *SmaI* PFGE profiles, whereas 6 strains were classified as non-typeable (NT) and were excluded from comparative PFGE pattern analysis. A similarity coefficient (Sab) threshold of 0.722 (72.2%) was applied, resulting in the identification of 45 genotypes, labeled from A to AT, and 38 unique patterns (Un), representing 21.7% of all *S. aureus* strains. The most prevalent genotypes were AH (*n* = 10; 5.7%) and N (*n* = 9; 5.1%). The remaining genotypes comprised two to five strains each. Genotypes V, W, and Z included five strains each (2.9%); AC, AM, and AN included four strains each (2.3%); and E, G, M, R, S, T, U, AB, AO, AP, and AS included three strains each (1.7%). Other genotypes (A, B, C, D, F, H, I, J, K, L, O, P, Q, X, Y, AD, AE, AF, AG, AI, AJ, AK, AL, AQ, AR, and AT) included two strains each (1.1%). In almost every genotype, the software identified 2 to 10 subtypes consisting of individual strains with similar but non-identical restriction profiles. An exception was genotype AD, represented by two strains with identical restriction patterns. In six *S. aureus* strains (3.4%), *SmaI* did not digest genomic DNA, and these were classified as non-typeable (NT). The macrorestriction analysis of nasal strains from students of a medical university is presented in the dendrogram (Figure 1).

In addition, a detailed analysis of individual genotypes and their PFGE subtypes revealed marked diversity, with various combinations of virulence genes and resistance phenotypes, even among strains belonging to the same genotype (Supplementary Materials—Table S4). For instance, genotype K showed substantial heterogeneity: two strains (subtypes K1 and K2, isolated from different study programs) carried the same hemolysin genes (*hla*, *hld*, *hlg*), but differed in *agr* group affiliation (3 vs. 1), enterotoxin profiles (*g i m o u* vs. *sea*, *sep*), and resistance phenotypes (K1—susceptible; K2—resistant to E, CC—iMLS_B_). Comparable differences were also observed in genotypes B, D, G, I, O, S, T, AE, AF, and AG.

In contrast, some genotypes displayed uniformity, with subtypes sharing the same *agr* groups, virulence profiles, and resistance phenotypes. These strains were either derived from students in the same field (e.g., C, L, P, AD) or from students in different fields (e.g., E, M, R, Z, AC, AQ). The largest genotype, AH, comprised 10 strains (all in *agr* group 3) that were subdivided into distinct PFGE subtypes. Eight *S. aureus* strains carried the *tst* gene but differed in enterotoxin content and originated from five different programs. The second most prevalent genotype, N, included nine strains, of which four (representing four subtypes) exhibited identical gene profiles and were all isolated from second-year medical students. Within the same genotype, two strains (positive for the *agr-1* gene) were obtained from students of different programs: second-year medicine and first-year nursing. Similar variability was noted among strains with unique restriction patterns. Some carried gene combinations absent from PFGE-defined genotypes; e.g., strain Un20 (from a 2nd-year paramedic student) harbored the *lukS-PV*/*lukF-PV* gene. Others shared gene profiles with established genotypes (e.g., Un5, Un32, Un38—AQ; Un8—L; Un21, Un22, Un26—M, Z; Un36, Un37—AD) or subtypes (e.g., Un5, Un32, Un38—E2, AC1, AC2; Un7, Un14, Un35—H1, J2, N3, N5, U1, U2, AG2, AS1, AS2).

All non-typeable strains differed in their distributions of virulence genes and antibiotic resistance profiles. Nevertheless, some exhibited gene profiles identical to those observed in unique strains (e.g., NT2—Un8, NT3—Un36, Un37), as well as in defined genotypes (e.g., NT2—L, NT3—AD) or subtypes (e.g., NT2—S2, AH2, AH10, AI1, AN1; NT3—A2, E1, E3; NT6—J1).

## 3. Discussion

In our study involving 800 students, *S. aureus* nasal carriage was confirmed in 175 individuals (21.9%), including 52 men and 123 women. Statistical analysis showed no significant association between sex and carriage. Similar findings were reported by Nilsson and Ripa [24], who also found no significant difference in carriage rates by sex among young adults. In contrast, other studies, such as those by Kluytmans et al. [9] and Gorwitz et al. [25], reported higher nasal carriage rates among men. These discrepancies may be attributed to differences in population characteristics, lifestyle factors, environmental influences, or diagnostic methods used. Our findings contribute to the growing body of evidence suggesting that the relationship between sex and *S. aureus* carriage is not straightforward and may depend on various contextual factors.

In the epidemiology of staphylococcal infections, MRSA carriage plays a significant role. Its spread among ambulatory patients is increasing and remains challenging to control. Studies by Choi et al. [14] and Kildow et al. [26] reported MRSA nasal carriage rates among adults ranging from 0.3% to 3.2%, respectively, whereas Chmielowiec-Korzeniowska et al. [17] found no MRSA strains among isolates obtained from healthy individuals. MRSA carriage is most commonly observed in individuals sharing the same habitat, such as nursing home residents, healthcare workers, sports team members (e.g., football players), participants in contact sports (e.g., wrestling or boxing), prisoners, and military personnel [27].

The MRSA carrier state poses an epidemiological concern in a hospital environment due to the risk of transmission. Carriers spread among healthcare personnel increase this risk to patients, especially those after surgery, immobilized, and with coexisting comorbidities. Nevertheless, the phenomenon of carrier state transmission operates in both directions, with health care staff becoming colonized by MRSA strains from colonized patients. Albrich and Harbarth [28] performed a meta-analysis of publications from 1980 to 2006 on the carriage of MSSA and MRSA strains among medical personnel (mainly nurses) from Europe, North and South America, Africa, Asia, and Australia and New Zealand. Among the approximately 33,000 participants in the study, nasal vestibular carriage of MSSA and MRSA was detected in 23.7% and 4.6% of healthcare workers, respectively. Similar results were obtained in Poland, where 26.0% of nurses, nurse practitioners, and midwives (for MSSA) and 4.0% (for MRSA) were colonized with *S. aureus* in the nasal vestibule [29]. Recent studies from the last five years consistently report low to moderate MRSA carriage rates among medical students, even in cohorts with regular clinical exposure [21,22,23]. These findings suggest that MRSA colonization in this population is primarily sporadic and influenced more by community-associated strains than hospital acquisition. The current literature further emphasizes the importance of continuous surveillance of asymptomatic carriers in educational healthcare settings, as highlighted in recent epidemiological studies and systematic reviews.

*S. aureus* nasal carriage also indicates students of medical faculties. Due to the study program, which includes practical classes in various departments and hospitals, constant contact with staff, patients, and the hospital environment can contribute to colonization with hospital strains, including MRSA. The presence of *S. aureus* in the nasal vestibule of students of medical universities was examined by other authors, who reported carriage rates ranging from 16.8% to 38.3% and MRSA prevalence between 1.9% and 9.4% [30,31,32,33,34]. Interestingly, in nursing students, *S. aureus* carriage was reported in 21.7% in Brazil [35] and 37.0–62.0% in Portugal [36], with MRSA prevalence ranging from 0% to 25% of the population. The above-mentioned literature indicates that the prevalence of *S. aureus* (including MRSA strains) carrier state varies among students from different faculties and years of study.

In the current cross-sectional study, the *S. aureus* carrier state rate was evaluated among students of different faculties and years of study at PMU in Szczecin, Poland. Students of medicine, dentistry, nursing, midwifery, and paramedic practice in hospitals, whilst students of medical analytics, medical biotechnology, and dietetics had no contact with a hospital department. Nasal vestibule swabs were collected from 800 students. *S. aureus* was found in 175 of them (21.9%). The lowest carriage rate was observed in second-year medical biotechnology students (9.1%), and the highest in fifth-year medical analytics students (26.9%). No MRSA strains were found. Similar results were obtained by Treesirichod et al. [37] and Trépanier et al. [38], who detected only MSSA strains in students without prior hospital contact.

*S. aureus* strains isolated from community patients showed better drug susceptibility than strains from the hospital environment, and these results apply to strains from both carriers and infections. Other studies conducted in our department confirmed overall good drug susceptibility: 84.3% among patients with boils [39] and in patients with atopic dermatitis [40].

In our study, the susceptibility of all strains to SXT, CIP, E, CC, and MUP was determined. The majority of *S. aureus* strains (84.0%) were susceptible to all tested drugs. Only a few strains showed resistance: SXT (0.6%), CIP (0.6%), and MUP (1.7%). Comparable results were reported by Adesida et al. [41], who observed no resistance to CIP or MUP among 26 isolates from 185 medical students, and by Budri et al. [42], who found 100% susceptibility to SXT and only one MUP-resistant strain (0.7%) among 444 Irish medical students without recent hospital exposure. Khashei et al. [43] reported four resistant strains to SXT among 40 MSSA isolates from 200 first-year medical students, while no resistance to CIP or MUP was found. Similarly, Baek et al. [44] found no SXT-resistant strains among dental students, whereas Okamo et al. [45] detected CIP-resistant isolates among second- and fifth-year medical students.

Resistance to E and CC among PMU students was low (13.7% each). By contrast, Du et al. [46] reported higher resistance rates among Chinese medical students (46.6% for E; 25.9% for CC), whereas Demirel et al. [47] found lower values in Turkey (6.0% and 4.5%, respectively). Among *S. aureus* strains from PMU students, the predominant resistance phenotype was iMLS_B_, detected in 12.6% of strains, while the cMLS_B_ phenotype was identified in two isolates. Treesirichod et al. [37] reported a much higher prevalence (62.3%) of iMLS_B_ among third-year medical students. Similarly, Saha and Krishnamurthy [48] confirmed iMLS_B_ in 11.4% of nursing and pharmacy students in India.

*S. aureus* carrier state plays an important role in the emergence of *S. aureus* and therefore contributes to infections. *S. aureus* shows some cellular and extracellular factors responsible for virulence and invasiveness. The expression of virulence genes varies and determines the disease outcome and clinical picture. In this study, we analyzed genes encoding hemolysins (*hla-hlg*), TSST-1 (*tst*), PVL (*lukS-PV*/*lukF-PV*), ETs (*eta*, *etb*, *etd*), SEs (*sea-seu*), and *agr* (*agr-1*–*agr-4*).

The gene encoding hemolysin α was found in all isolates. Genes encoding δ- and γ-hemolysins were present in most strains (93.7% and 93.1%), but less frequently in paramedic (*hld*—41.7%) and first-year nursing students (*hlg*—73.3%). Other authors reported similar results for *hla*, *hld*, and *hlg* (97.7–100%) [49,50,51]. The prevalence of the *hlb* gene reported by others (43.7–98.0%) was higher than in our study. The *hlb* was detected only in 5.3–16.7% of strains from fifth-year medical analytics, third-year medicine, second-year dentistry, and second-year paramedic students. Strains producing β-hemolysin exhibit characteristic double hemolysis on blood media but are less frequently isolated from humans, as confirmed by our study. β-hemolysin is mainly produced by strains isolated from household animals [52].

The *tst* gene, encoding TSST-1, was present in 22.3% of isolates, ranging from 6.7% to 66.7% across faculties, and was absent in only six strains from first-year midwifery students. Collery et al. [53] reported lower percentages (12.5%) among medical, dental, nursing, and dental hygiene students. Similar results (24.3%) were obtained by Mehrotra et al. [54] in healthy nasal carriers, and by Piechowicz et al. [33], who found *tst* in 6.7% of first-year medical students before clinical practice and 21.6% of third-year students during clinical training.

In our study, the *tst* gene was detected in 39 isolates, of which 36 (the vast majority) carried an *egc* operon. The *tst* gene occurred significantly more often in combination with the *egc* cluster variants, including *g i m o u* and *g i o u*, which were the only clusters showing a statistically significant association with *tst* in our dataset. These findings are consistent with observations reported by German researchers, who also described an association between *tst* and extended *egc* cluster combinations, including *g i m n o u*, *g i m o u*, and *g i o u* [55]. Our results therefore support the concept that *tst*-positive *S. aureus* strains are predominantly linked to *egc*-positive genetic backgrounds, particularly those harboring the *g i m o u* or *g i o u* cluster variants. Additionally, the *tst* gene was significantly more frequent with the *agr-3* gene (*p* < 0.001), consistent with previously published data confirming the association of *agr-3* with *tst* [56,57,58,59].

*S. aureus* strains carrying the *lukS-PV*/*lukF-PV* gene (which encodes PVL) are most often isolated from patients suffering from skin and subcutaneous tissue infections, as well as boils [60]. It was demonstrated, among other things, by a study by Masiuk et al. [39], which found that 85.0% of *S. aureus* strains from patients with boils carried this gene. The *lukS-PV*/*lukF-PV* gene is rarely detected among *S. aureus* isolated from carriers (e.g., students). It is more frequently found among MRSA strains. It was demonstrated, among others, by the study by Bettin et al. [61], which found the gene encoding PVL in 4 (4.2%) of 95 MSSA isolates and in 5 (83.3%) of 6 MRSA isolates from medical students. In contrast, Abroo et al. [62] found the *lukS-PV*/*lukF-PV* gene only in 1 (5.6%) MRSA strain from a medical student. In the present study, no MRSA strains were detected, while one (0.6%) MSSA isolate from a second-year paramedic student carried the *lukS-PV*/*lukF-PV* genes.

ETs are important virulence factors of *S. aureus*, though the genes encoding them are rare among carriers. Literature data indicate that, in hospitalized patients, the *eta* and *etb* genes are found in 1–2% of isolates [59,63], whereas Rasmussen et al. [64] reported 6.5% *eta*- and *etd*-positive strains, but none were *etb*-positive. Among healthy volunteers, Abimanyu et al. [63] detected *eta* and *etb* in 2.3% and 0.6% of strains, respectively, with no *etd*. These findings are consistent with the current study, which identified the *eta* gene in 1.1% of isolates (dentistry and nursing students) and the *etd* gene in 1.7% (dietetics and medical students), while *etb* was absent.

The *egc* operon, located on the vSAβ genomic island [55], is frequently detected in *S. aureus* strains isolated from healthy carriers (e.g., outpatients, inpatients, and students), with prevalence ranging from 47.0% to 87.8% [42,58,64,65]. In our study, the prevalence of *egc*-positive isolates from the nasal vestibule of students at PMU in Szczecin was 53.7%. The most commonly identified genes were *seg* (53.7%), *sei* (53.7%), *seo* (53.7%), and *sem* (44.6%), while *seu* (29.7%) and *sen* (25.7%) were less frequent. Mendoza et al. [66], analyzing samples from healthy volunteers, detected *seg*, *sei*, *sem*, *sen*, and *seo* in 76.7% of isolates, and *seu* in 39.5%. In contrast, Nashev et al. [67] reported *seg*, *sei*, *sem*, and *sen* in 73.4% of strains, *seo* in 66.7%, and *seu* in 71.1% of isolates. Thus, the distribution of *egc* cluster genes may vary between different populations and regions. Importantly, these genes rarely occur alone but rather form defined combinations within the *egc* cluster. Two main variants are distinguished: *egc1*, consisting of *seg*, *sei*, *sem*, *sen*, and *seo* (*g i m n o*), and *egc2*, which additionally includes *seu* (*g i m n o u*). Such gene arrangements are most often observed in carrier strains, though with varying frequencies. For example, Bania et al. [64] reported a predominance of *egc1* (45.9%) over *egc2* (31.1%) among 61 strains isolated from the nasal vestibule of hospitalized patients.

Among the other SEs tested, the gene encoding the SEP toxin was found in 17.1% of the strains. This protein, encoded by a prophage (Sa3n), is classified as a SAg [68,69]. Other studies report a lower percentage (1.6%) of the *sep* gene among carrier-derived *S. aureus* strains [64]. In our study, the *sep* gene was present in 29 of 81 (35.8%) strains lacking the *egc* cluster, which accounted for 96.7% of all *sep*-positive isolates (*p* < 0.001). Similar results were obtained by Aydin et al. [70], who found that 15 of 17 *sep*-positive isolates lacked *egc* cluster genes. Our study also showed that the presence of the *sep* gene statistically significantly correlates with the *agr-1* gene (*p* < 0.001).

The prevalence of the *sea* gene among *S. aureus* strains isolated from nasal carriers reported in the literature ranges from 8.8% to 26.0% [53,55,64,67,71]. A similar proportion (13.7%) was observed in our study. The presence of the *sea* gene showed only borderline statistical significance with the *g i m o u* cluster variant (*p* = 0.05), suggesting a weak association rather than an explicit confirmation. In contrast, a statistically significant relationship was observed between the *sea* and *agr-3* genes (*p* = 0.026), with *sea* occurring more frequently. This finding is consistent with observations by Holtfreter et al. [55], who demonstrated that *sea*-positive strains most commonly belong to *agr-3*, although they reported a stronger association with extended *egc* cluster combinations. Overall, our results suggest that the distribution of the *sea* gene in the studied population is relatively uniform, although it may be influenced by the regulatory background associated with the *agr-3* group.

The *sec* and *sel* genes were detected less frequently among the strains tested, at 7.4% each, although the *sel* gene was carried by up to 92.3% of *sec*-positive isolates (*p* < 0.001). In slightly higher percentages (12.4–19.7%) and always simultaneously, these genes were also found by other authors [55,64,65]. In addition, Bania et al. [64] showed that among *sec–sel*-positive strains, the *egc1* cluster was also detected. In the present study, both the *sec* (*p* < 0.001) and *sel* (*p* < 0.001) genes occurred significantly more frequently not only within the *g i m n o* cluster but also in isolates carrying the *agr-1* gene, which is consistent with earlier observations linking *sec*, *sel*, *egc1*, and *agr-1*. Holtfreter et al. [55] likewise noted that *sec* and *sel* were detected more often in strains harboring *egc1* and belonging to *agr-1*.

According to the available literature, the genes encoding the remaining examined SEs (*seb*, *sed*, *see*, *seh*, *sej*, *sek*, *seq*, and *ser*) are detected at low frequencies, not exceeding 10%, or are absent among *S. aureus* strains isolated from nasal carriers [64,65,67,71]. Similar results were obtained in our study (0.6–2.3%), and the *see* gene was not detected in any isolate. It is known that some of these genes are located on a shared mobile genetic element, which determines their characteristic co-occurrence patterns. For example, the *sed*, *sej*, and *ser* genes are carried together on the pIB485-type plasmid [72], and the regulatory region of the *sed* gene is under the control of the *agr* operon, which may additionally influence their expression.

It is also noteworthy that a significant association was observed between isolates positive for the *agr-3* and *seh* genes (*p* = 0.006), suggesting that *agr*-dependent regulation may influence the distribution of less common enterotoxin genes. Similar findings were reported by Sapri et al. [73], who also demonstrated association between the *agr-3* group and the *seh* gene.

Accordingly, analysis of the relationship between the occurrence of individual *egc* clusters and the presence of virulence genes revealed statistically significant associations between specific gene combinations and SAg profiles. In the *g i m n o* cluster, the *sed* (*p* = 0.012), *sej* (*p* = 0.012), and *ser* (*p* = 0.012) genes were detected significantly more frequently, which is consistent with their shared localization on the pIB485 plasmid. These findings suggest that, despite their overall low prevalence, plasmid-encoded SE genes tend to cluster in strains carrying specific *egc* operon variants, supporting the concept of coordinated genetic mobility and conserved virulence-gene arrangements in *S. aureus*.

*S. aureus* virulence factors expression is under the control of the global gene expression regulation (*agr*) system, primarily conditioned by a high cell density [74,75]. In our study, among isolates from the nasal vestibule of students of a medical university, *agr-1* (49.7%) predominated, followed by *agr-3* (28.6%) and *agr-2* (20.0%); only three (1.7%) strains harboured *agr-4*. Similar results were described by Peacock et al. [76] in strains from healthy individuals, with *agr-1* and *agr-3* predominant (37.0%), followed by *agr-2* (24.0%) and *agr-4* (3.0%). Rasmussen et al. [65] also showed that the most common *agr* genes among hospitalized patients were *agr-1* (46.0%) and *agr-3* (39.0%), while *agr-2* (11.0%) and *agr-4* (4.0%) were less frequent. Collery et al. [53] reported slightly different results among medical and dental hygiene students: *agr-3* (37.5%), followed by *agr-1* (35.4%), *agr-2* (25.0%), and only 2.1% for *agr-4*.

In a study by Masiuk et al. [39] on *S. aureus* strains from boils, a correlation was found between *agr-1* and *egc1*, and between *agr-4* and *egc2*. Similarly, in our study, *egc1* was also identified within the *agr-1* group, and this relationship was confirmed as statistically significant (*p* < 0.001). The *egc2* cluster was detected in four isolates, three of which belonged to *agr-4*, demonstrating a significant association with this *agr* group (*p* < 0.001). The findings reported by Holtfreter et al. [55], who observed that *agr-3* strains most commonly harbor *egc* operon genes in combination with *egc2*, were not confirmed in our dataset. Instead, a significant association was identified between *agr-3* and the *g i m o u* and *g i o u* clusters (both *p* < 0.001).

In monitoring the transmission of *S. aureus* in the environment (carrier-patient infection), an important role is played by the potential genetic relatedness of the strains, determined by various genetic techniques. Such techniques include PFGE, which is considered the “gold standard” for molecular typing of microorganisms [77]. In the present study, 45 PFGE genotypes and 38 unique restriction patterns were described among 169 strains isolated from PMU students. Six isolates were classified as non-typeable (NT) and therefore were not included in the comparative analysis of PFGE patterns. Within each genotype, 2–10 subtypes were also distinguished. Only in one AD genotype were two strains found to have identical restriction patterns [78,79,80].

The number of genetic types, unique PFGE patterns, and NT strains of *S. aureus* observed in isolates from students of each faculty varied, although the number of strains obtained was very similar. The PFGE clustering was interpreted based on Dice coefficient similarity using UPGMA analysis, with a 72.2% similarity threshold applied in accordance with commonly used criteria for defining genetic relatedness in *S. aureus* epidemiological studies. This approach is consistent with previously published PFGE-based investigations, where similarity cut-offs in the range of 70–80% are routinely used to delineate clonal relationships and assess population structure.

These results revealed low relatedness among *S. aureus* strains colonizing the students and indicated the lack of single dominant pattern. Similar results were also observed by Adesida et al. [41], who examined 185 third-year medical students and found *S. aureus* in 26 individuals (14.1%). PFGE determined genetic relatedness analysis of 16 strains with *SmaI*, and *S. aureus* was divided into seven genotypes (A–G). In contrast, El-Mahdy et al. [81] classified 21 strains from carriers into 12 genetic types (A–L).

The vast diversity of *S. aureus* strains isolated from colonized students was demonstrated by detailed analysis of PFGE genotypes and their subtypes, as reflected in the occurrence of combinations of genes encoding virulence factors and resistance phenotypes. Strains within different subtypes of the same genotype, for instance, showed the presence of the same hemolysin genes but differed in *agr* genes, enterotoxin genes, or resistance phenotype. In contrast, other subtypes showed the same *agr* groups, virulence genes, and resistance phenotypes. Similar variation was observed among strains with unique restriction patterns and among NT strains. Moreover, the absence of MRSA may reflect effective infection control practices, structured clinical training, and the predominantly community-acquired nature of nasal *S. aureus* carriage in young healthy individuals [28,82].

## 4. Conclusions

In conclusion, this cross-sectional study confirmed the high genetic variability of *S. aureus* colonizing students of various faculties and years at PMU in Szczecin, Poland. The results demonstrate the considerable diversity of this species and its ability to adapt to environmental conditions. The analyzed isolates exhibited considerable genetic diversity without evidence of widespread dissemination of a predominant epidemic pulsotype. The carrier rate was similar to that reported in the general outpatient population (21.9%) and did not differ by field or year of study. Most isolates showed good susceptibility to antimicrobial agents, with 84.0% susceptible to all tested drugs, including mupirocin used for the eradication of the carrier state, and importantly, the lack of methicillin resistance among strains was confirmed. The absence of MRSA in this cohort is consistent with current infection control data indicating low MRSA carriage rates among medical students despite regular clinical exposure. This finding may be explained by the predominantly community-acquired nature of nasal carriage in young healthy individuals and limited sustained exposure to high-risk hospital environments. Similar recent studies have also reported low or absent MRSA carriage among medical students in European populations despite clinical training exposure.

The study is limited by its cross-sectional design, single-center setting, and focus on a specific student population, which may limit the generalizability of the findings to other groups or healthcare settings.

The diversity of virulence genes, the *agr* system, and PFGE restriction patterns revealed the heterogeneity of the strains and the absence of epidemic spread among colonized students. However, further research is needed to understand better the dynamics and persistence of *S. aureus* strains circulating among young adults. Moreover, genomic approaches may help to clarify the evolutionary mechanisms underlying the observed diversity. From a practical perspective, the findings highlight the importance of awareness of asymptomatic nasal carriage among medical students and support the reinforcement of infection prevention measures, including hand hygiene and adherence to clinical safety protocols during healthcare training.

## 5. Limitations of the Study

This study has several limitations that should be considered when interpreting the results. The isolates included in the analysis were collected in 2014–2015, and although all strains were stored at –80 °C to preserve their genetic integrity, the findings may not fully reflect the current epidemiological situation. However, the primary aim of the study was to investigate relatively stable genetic characteristics of *S. aureus*, such as *egc* cluster variants, *agr* genes, and selected virulence-associated genes, rather than to describe contemporary epidemiological trends.

The study was conducted among young adults from a single medical university, which may limit the generalizability of the findings to other populations or settings. Additionally, some student subgroups were represented by only a few isolates, which may reduce the statistical power to detect subtle differences in gene distribution.

The analysis of virulence determinants focused solely on the presence of selected genes, without assessing their expression or functional activity. Similarly, although antimicrobial susceptibility testing was performed, it included only a selected panel of commonly used antibiotics and did not involve minimum inhibitory concentration determination or molecular characterization of resistance mechanisms. As a result, the full spectrum of resistance mechanisms and their potential associations with virulence genotypes could not be fully evaluated. PFGE was used as the primary typing method; although traditionally regarded as the gold standard in epidemiological investigations, it offers lower resolution than whole-genome sequencing, which could provide more detailed information on strain-relatedness and genetic diversity.

Despite these limitations, the study provides valuable insights into the genetic characteristics of nasal *S. aureus* colonization in healthy young adults, including the distribution of *egc* cluster variants, *agr* groups, virulence genes, and antimicrobial resistance profiles.

## 6. Materials and Methods

### 6.1. Study Population

This cross-sectional study was conducted between October 2014 and December 2015 as part of practical laboratory classes at the Chair of Microbiology and Immunology (currently Chair of Microbiology, Immunology, and Laboratory Medicine) of the PMU in Szczecin, Poland.

A total of 800 students (223 men and 577 women) participated in the study from 11 various faculties: medical analytics, 3rd year (MA3; *n* = 31); medical analytics, 5th year (MA5; *n* = 26); medical biotechnology, 2nd year of first-cycle (MB2; *n* = 33); dietetics, 1st year (D1; *n* = 63); medicine, 2nd year (MED2; *n* = 210); medicine, 3rd year (MED3; *n* = 167); dentistry, 2nd year (DENT2; *n* = 92); nursing, 1st year (NUR1; *n* = 57); midwifery, 1st year (MID1; *n* = 37); midwifery, 2nd year (MID2; *n* = 36); paramedic, 2nd year (PAR2; *n* = 48). All participants provided informed consent to take part in the study. Inclusion criteria comprised students who voluntarily participated in the screening and provided informed consent. Exclusion criteria included antibiotic use within six months prior to sampling, and students reporting recent antimicrobial therapy were excluded from the study.

The study was conducted in accordance with the Declaration of Helsinki and was approved by the Ethics Committee of PMU (protocol code KB-0012/04/01/14, date 15 January 2015) for studies involving humans.

### 6.2. Isolation and Identification of Bacterial Strains

Swab collection and bacterial identification were performed according to standard microbiological diagnostic procedures. To detect the presence of *S. aureus*, a nasal vestibule swab was taken from each student. All samples were cultured on Columbia agar with 5% sheep blood and Chapman agar (bioMérieux, Warsaw, Poland) and incubated for 18 h at 37 °C. Identification of *S. aureus* species was based on mannitol fermentation, rabbit plasma coagulase activity, and biochemical properties using the VITEK2 Compact system (bioMérieux, Warsaw, Poland). Additionally, amplification of the *nuc* gene was carried out using a single PCR assay.

### 6.3. Antimicrobial Susceptibility Testing (AST)

The AST of *S. aureus* strains to selected drugs was assessed using the disc diffusion method, in accordance with the European Committee on Antimicrobial Susceptibility Testing recommendations [83]. The following antibiotic discs were used: MUP (200 µg), CIP (5 µg), SXT (1.25/23.75 µg), E (15 µg), CC (2 µg), and FOX (30 µg—used to detect MRSA phenotype) (Becton Dickinson, Warsaw, Poland). Briefly, the strains were cultured on Columbia agar with 5% sheep blood and incubated aerobically at 37 °C for 18 h. A bacterial suspension with a turbidity of 0.5 on the McFarland scale was then prepared. This suspension was inoculated onto Mueller-Hinton agar (bioMérieux, Warsaw, Poland), and antibiotic-impregnated discs were applied to the surface. The MLS_B_ resistance phenotype was determined using the D-test method, with E and CC discs placed 12–20 mm apart (edge-to-edge) [84]. Resistance to both antibiotics indicated a cMLS_B_ phenotype. Resistance to E, along with a D-shaped inhibition zone around the CC disc, indicated an iMLS_B_ phenotype. Resistance to E and concurrent susceptibility to CC indicated the presence of the MS_B_ resistance mechanism.

### 6.4. Molecular Biology Methods

The study employed PCR-based techniques (Single PCR and Multiplex PCR) and pulsed-field gel electrophoresis (PFGE). Genomic DNA extracted from the isolates was used to detect the *nuc* (encoding thermonuclease), *tst* (encoding TSST-1), *lukS-PV*/*lukF-PV* (encoding the two subunits of PVL), *hla–hlg* (encoding hemolysins), *eta* (encoding ETA), *etd* (encoding ETD), *sea–seu* (encoding SEs), and *agr-1*–*agr-4* (encoding accessory gene regulator [*agr*] system involved in the regulation of virulence factor expression) genes. Plasmid DNA was used to detect the *etb* gene (encoding ETB).

#### 6.4.1. Isolation of Genomic and Plasmid DNA

Genomic and plasmid DNA were isolated for downstream molecular analyses using commercial kits. To prepare the bacterial material for extraction, a single colony of each strain from an 18 h culture on Columbia agar with 5% sheep blood was inoculated into 3 mL of tryptic soy broth and incubated for 18 h at 37 °C. Following incubation, DNA was isolated using the GeneMATRIX Bacterial & Yeast Genomic DNA Purification Kit and the GeneMATRIX Plasmid Miniprep DNA Purification Kit (EURx, Gdansk, Poland), in accordance with the manufacturer’s instructions. The resulting purified DNA samples were stored at –20 °C. DNA concentration and purity were assessed using a NanoDrop 1000 Spectrophotometer (Thermo Fisher Scientific, Wilmington, DE, USA).

#### 6.4.2. Single PCR Assay

Amplification of the *nuc*, *lukS-PV*/*lukF-PV*, *hla–hlg*, and *etb* genes was performed using the StartWarm HS-PCR Mix (A&A Biotechnology, Gdansk, Poland), which contains: 2.5 mM MgCl_2_, 0.5 mM of each dNTP (dATP, dCTP, dGTP, dTTP), PCR buffer, Taq DNA polymerase (0.1 U/µL), red dye, and loading buffer. Each 25 µL PCR reaction mixture consisted of: 12.5 µL StartWarm HS-PCR Mix, 0.75 µL of each primer (forward and reverse) (Institute of Biochemistry and Biophysics, Polish Academy of Sciences, Warsaw, Poland) at a final concentration of 150–400 nM, 10 µL of nuclease-free water (Thermo Fisher Scientific, Waltham, CA, USA), and 1 µL of template DNA (10–20 ng/µL). Product sizes and primer sequences used in the Single PCR assays are listed in Supplementary Table S1.

Single PCR assay was carried out in an Applied Biosystems Veriti 96 Well Thermal Cycler (Applied Biosystems, Norwalk, CT, USA) under the following conditions: initial denaturation—4 min at 95 °C, 30 cycles of denaturation—30 s at 95 °C, primer annealing—30 s at 55 °C, and elongation—1 min at 72 °C followed by a final extension step—10 min at 72 °C. Amplified products were separated by electrophoresis on a 1.5% agarose gel (DNA Gdansk, Poland) stained with ethidium bromide (0.5 µg/mL; Sigma-Aldrich, Darmstadt, Germany), in 1×TRIS/boric acid/EDTA (TBE buffer; Thermo Fisher Scientific, Waltham, CA, USA) for 60 min at 25 °C and 100 V. A 100 bp DNA ladder (DNA Gdansk, Gdansk, Poland) was used as a molecular weight marker (range: 100–1000 bp). Control strains carrying the respective target genes were included in each assay and are listed in Supplementary Table S1. Each PCR run included a negative control (reaction mixture without template DNA). Amplified products were visualized under UV light using the GelDoc-It^2^ Imager system (Upland, CA, USA). All amplifications were performed in duplicate to ensure reproducibility.

#### 6.4.3. Multiplex PCR Assay

Multiplex PCR was used to amplify fragments of the following genes: *sea*, *seb*, *sec*, *sed*, *see*, *seg*, *seh*, *sei*, *sej*, *sek*, *sel*, *sem*, *sen*, *seo*, *sep*, *seq*, *ser*, *seu*, *tst*, *eta*, and *etd*, as well as genes of the *agr* system (*agr-1*–*agr-4*). Six different Multiplex PCR sets (I–VI) were prepared for this purpose. Primer sequences, the sizes of the amplified products, and the control strains used in each assay are listed in Supplementary Table S2.

The Multiplex PCR reactions were carried out using 5×Green GoTaq^®^ Reaction Buffer (Promega, Madison, WI, USA), 10 mM dNTP mix (Promega, Madison, WI, USA), 25 mM MgCl_2_ (Promega, Madison, WI, USA), primer pairs at a final concentration of 150–400 nM each (Institute of Biochemistry and Biophysics, Polish Academy of Sciences, Warsaw, Poland), Hot-Start DNA polymerase at a concentration of 1.0 U/µL (A&A Biotechnology, Gdansk, Poland), nuclease-free water (Thermo Fisher Scientific, Waltham, CA, USA), and 1 μL of template DNA (10–20 ng/μL). Detailed reagent volumes are listed in Supplementary Table S3.

The PCR cycling conditions for Multiplex PCR were similar to those used in the Single PCR assays. Likewise, the electrophoretic separation of the amplified products was performed under the same conditions, using a 1.5% agarose gel stained with ethidium bromide (0.5 µg/mL) in 1×TBE buffer, at 100 V for 90 min at 25 °C. A 100 bp DNA ladder was used as a molecular weight marker. Each Multiplex PCR reaction was carried out in duplicate to ensure reproducibility of the results.

### 6.5. Genetic Relatedness Analysis of S. aureus Strains by the PFGE Method

#### 6.5.1. Isolation of Genomic DNA and Restriction Digestion

Before DNA isolation, the tested *S. aureus* strains were thawed, streaked onto Columbia agar with 5% sheep blood agar plates, and incubated at 37 °C for 18 h. A single colony was then transferred to 3 mL of TSB and cultured for another 18 h. From this culture, 1 mL of the bacterial suspension was collected, centrifuged (5000× *g*, 5 min), and the supernatant was discarded. The resulting cell pellet was used for genomic DNA isolation. The procedure was performed according to the GenePath Group 6 Reagent Kit Instruction Manual (Bio-Rad, Marnes-la-Coquette, France) using the CHEF Bacterial Genomic DNA Plug Kit (Bio-Rad, Marnes-la-Coquette, France). Briefly, the pellet was suspended in Cell Suspension Buffer and then treated with lysozyme (0.025 g/mL) and lysostaphin (400 U/mL) (DNA Gdansk, Gdansk, Poland). The mixture was combined with melted 2% agarose (at 55 °C), forming agarose plugs containing genomic DNA. After solidification, the plugs were incubated in lysis buffer with lysozyme and lysostaphin at 37 °C for 2 h, followed by digestion in proteinase K buffer at 50 °C for 20 h. After incubation, the plugs were washed three times and stored in Wash Buffer (1×, Bio-Rad, Marnes-la-Coquette, France) at 4 °C.

Prior to analysis, the plugs were digested with the restriction enzyme *SmaI* (Thermo Fisher Scientific, Waltham, CA, USA) in Tango buffer (Thermo Fisher Scientific, Waltham, CA, USA) for 20 h at 30 °C. After digestion, the plugs were washed four times and stored in Wash Buffer (1×) at 4 °C until further analysis.

#### 6.5.2. PFGE Method

Genetic relatedness among the *S. aureus* strains was assessed by PFGE using the CHEF DR^®^ III system (Bio-Rad, Marnes-la-Coquette, France). DNA fragments were separated in a 1.2% agarose gel prepared in 1× TBE buffer and run at 14 °C under the following conditions: run time—22 h; initial switch time—2.2 s; final switch time—54.2 s; angle—120°; voltage—6 V.

After electrophoresis, gels were stained with ethidium bromide (1:10,000 *v*/*v*), rinsed in deionized water, and visualized under UV light using the GelDoc-It^2^ Imager (Upland, CA, USA). Restriction profiles were analyzed using FPQuest software (Bio-Rad, Marnes-la-Coquette, France). Genetic similarity was evaluated using the unweighted pair group method with arithmetic mean (UPGMA) and Dice coefficient with a 2% band position tolerance. The cut-off similarity value (Sab) was set at 72.2%. A dendrogram was constructed to illustrate the clonal relationships among the tested strains.

### 6.6. Statistical Analysis

Qualitative data were described using nominal and percentage values. Comparisons between groups were performed using chi-square test or Fisher’s exact test, as appropriate. Differences were considered statistically significant at *p* < 0.05.

## Figures and Tables

**Table 1:** Prevalence of *S. aureus* nasal carriage among students according to study.

Study	Students *n* = 800 ^1^	Carriers *n* = 175 ^1^	Non-Carriers *n =* 625 ^1^
MA3	31 (3.9)	8 (25.8)	23 (74.2)
MA5	26 (3.3)	7 (26.9)	19 (73.1)
MB2	33 (4.1)	3 (9.1)	30 (90.9)
D1	63 (7.9)	14 (22.2)	49 (77.8)
MED2	210 (26.3)	40 (19.0)	170 (81.0)
MED3	167 (20.9)	38 (22.8)	129 (77.2)
DENT2	92 (11.5)	24 (26.1)	68 (73.9)
NUR1	57 (7.1)	15 (26.3)	42 (73.7)
MID1	37 (4.6)	6 (16.2)	31 (83.8)
MID2	36 (4.5)	8 (22.2)	28 (77.8)
PAR2	48 (6.0)	12 (25.0)	36 (75.0)

^1^ *n* (%); MA3—medical analytics, 3rd year; MA5—medical analytics, 5th year; MB2—medical biotechnology, 2nd year of first-cycle; D1—dietetics, 1st year; MED2—medicine, 2nd year; MED3—medicine, 3rd year; DENT2—dentistry, 2nd year; NUR1—nursing, 1st year; MID1—midwifery, 1st year; MID2—midwifery, 2nd year; PAR2—paramedic, 2nd year.

**Table 2:** Prevalence of *S. aureus* nasal carriage among students by sex.

Study	Carriers		Non-Carriers	
	Male *n* = 52 ^1^	Female *n* = 123 ^1^	Male *n* = 171 ^1^	Female *n* = 454 ^1^
MA3	2 (25.0)	6 (75.0)	3 (13.0)	20 (87.0)
MA5	2 (28.6)	5 (71.4)	1 (5.3)	18 (94.7)
MB2	0 (0.0)	3 (100.0)	3 (10.0)	27 (90.0)
D1	0 (0.0)	14 (100.0)	8 (16.3)	41 (83.7)
MED2	14 (35.0)	26 (65.0)	75 (44.1)	95 (55.9)
MED3	18 (47.4)	20 (52.6)	42 (32.6)	87 (67.4)
DENT2	7 (29.2)	17 (70.8)	11 (16.2)	57 (83.8)
NUR1	1 (6.7)	14 (93.3)	2 (4.8)	40 (95.2)
MID1	0 (0.0)	6 (100.0)	4 (12.9)	27 (87.1)
MID2	0 (0.0)	8 (100.0)	4 (14.3)	24 (85.7)
PAR2	8 (66.7)	4 (33.3)	18 (50.0)	18 (50.0)

^1^ *n* (%); MA3—medical analytics, 3rd year; MA5—medical analytics, 5th year; MB2—medical biotechnology, 2nd year of first-cycle; D1—dietetics, 1st year; MED2—medicine, 2nd year; MED3—medicine, 3rd year; DENT2—dentistry, 2nd year; NUR1—nursing, 1st year; MID1—midwifery, 1st year; MID2—midwifery, 2nd year; PAR2—paramedic, 2nd year.

**Table 3:** *S. aureus* phenotypes identified based on antimicrobial resistance patterns according to study.

Characteristic	Total *n* = 175 ^1^	MA3 *n* = 8 ^1^	MA5 *n* = 7 ^1^	MB2 *n* = 3 ^1^	D1 *n* = 14 ^1^	MED2 *n* = 40 ^1^	MED3 *n* = 38 ^1^	DENT2 *n* = 24 ^1^	NUR1 *n* = 15 ^1^	MID1 *n* = 6 ^1^	MID2 *n* = 8 ^1^	PAR2 *n* = 12 ^1^
Antimicrobial status												
R	28 (16.0)	0 (0.0)	1 (14.3)	2 (66.7)	0 (0.0)	8 (20.0)	6 (15.8)	4 (16.7)	1 (6.7)	4 (66.7)	0 (0.0)	2 (16.7)
S	147 (84.0)	8 (100.0)	6 (85.7)	1 (33.3)	14 (100.0)	32 (80.0)	32 (84.2)	20 (83.3)	14 (93.3)	2 (33.3)	8 (100.0)	10 (83.3)
Resistance phenotype												
CIP	1 (0.6)	0 (0.0)	0 (0.0)	0 (0.0)	0 (0.0)	0 (0.0)	0 (0.0)	1 (4.2)	0 (0.0)	0 (0.0)	0 (0.0)	0 (0.0)
cMLS_B_	2 (1.1)	0 (0.0)	0 (0.0)	0 (0.0)	0 (0.0)	1 (2.5)	0 (0.0)	0 (0.0)	0 (0.0)	1 (16.7)	0 (0.0)	0 (0.0)
iMLS_B_	21 (12.0)	0 (0.0)	1 (14.3)	2 (66.7)	0 (0.0)	7 (17.5)	3 (7.9)	3 (12.5)	1 (6.7)	2 (33.3)	0 (0.0)	2 (16.7)
iMLS_B_ + MUP	1 (0.6)	0 (0.0)	0 (0.0)	0 (0.0)	0 (0.0)	0 (0.0)	1 (2.6)	0 (0.0)	0 (0.0)	0 (0.0)	0 (0.0)	0 (0.0)
MUP	2 (1.1)	0 (0.0)	0 (0.0)	0 (0.0)	0 (0.0)	0 (0.0)	2 (5.3)	0 (0.0)	0 (0.0)	0 (0.0)	0 (0.0)	0 (0.0)
SXT	1 (0.6)	0 (0.0)	0 (0.0)	0 (0.0)	0 (0.0)	0 (0.0)	0 (0.0)	0 (0.0)	0 (0.0)	1 (16.7)	0 (0.0)	0 (0.0)

^1^ *n* (%); R—resistance; S—susceptibility; CIP—ciprofloxacin; cMLS_B_—constitutive macrolide-lincosamide-streptogramin B (MLS_B_) resistance phenotype; iMLS_B_—inducible macrolide-lincosamide-streptogramin B (MLS_B_) resistance phenotype; MUP—mupirocin; SXT—trimethoprim/sulfamethoxazole; MA3—medical analytics, 3rd year; MA5—medical analytics, 5th year; MB2—medical biotechnology, 2nd year of first-cycle; D1—dietetics, 1st year; MED2—medicine, 2nd year; MED3—medicine, 3rd year; DENT2—dentistry, 2nd year; NUR1—nursing, 1st year; MID1—midwifery, 1st year; MID2—midwifery, 2nd year; PAR2—paramedic, 2nd year.

**Table 4:** The number and prevalence of genes encoding hemolysins, Panton-Valentine leukocidin (PVL), exfoliative toxins (ETs), toxic shock syndrome toxin-1 (TSST-1), and staphylococcal enterotoxins (SEs) among *S. aureus* strains isolated from the nasal vestibule of students according to study.

Genes	Total *n* = 175 ^1^	MA3 *n* = 8 ^1^	MA5 *n* = 7 ^1^	MB2 *n* = 3 ^1^	D1 *n* = 14 ^1^	MED2 *n* = 40 ^1^	MED3 *n* = 38 ^1^	DENT2 *n* = 24 ^1^	NUR1 *n* = 15 ^1^	MID1 *n* = 6 ^1^	MID2 *n* = 8 ^1^	PAR2 *n* = 12 ^1^
*hla*	175 (100.0)	8 (100.0)	7 (100.0)	3 (100.0)	14 (100.0)	40 (100.0)	38 (100.0)	24 (100.0)	15 (100.0)	6 (100.0)	8 (100.0)	12 (100.0)
*hlb*	9 (5.1)	0 (0.0)	1 (14.3)	0 (0.0)	0 (0.0)	0 (0.0)	2 (5.3)	4 (16.7)	0 (0.0)	0 (0.0)	0 (0.0)	2 (16.7)
*hld*	164 (93.7)	8 (100.0)	7 (100.0)	3 (100.0)	14 (100.0)	40 (100.0)	34 (89.5)	24 (100.0)	15 (100.0)	6 (100.0)	8 (100.0)	5 (41.7)
*hlg*	163 (93.1)	8 (100.0)	7 (100.0)	3 (100.0)	14 (100.0)	36 (90.0)	35 (92.1)	24 (100.0)	11 (73.3)	5 (83.3)	8 (100.0)	12 (100.0)
*lukS-PV*/*lukF-PV*	1 (0.6)	0 (0.0)	0 (0.0)	0 (0.0)	0 (0.0)	0 (0.0)	0 (0.0)	0 (0.0)	0 (0.0)	0 (0.0)	0 (0.0)	1 (8.3)
*eta*	2 (1.1)	0 (0.0)	0 (0.0)	0 (0.0)	0 (0.0)	0 (0.0)	0 (0.0)	1 (4.2)	1 (6.7)	0 (0.0)	0 (0.0)	0 (0.0)
*etd*	3 (1.7)	0 (0.0)	0 (0.0)	0 (0.0)	1 (7.1)	1 (2.5)	1 (2.6)	0 (0.0)	0 (0.0)	0 (0.0)	0 (0.0)	0 (0.0)
*tst*	39 (22.3)	2 (25.0)	1 (14.3)	2 (66.7)	4 (28.6)	5 (12.5)	13 (34.2)	6 (25.0)	1 (6.7)	0 (0.0)	3 (37.5)	2 (16.7)
*sea*	24 (13.7)	5 (62.5)	0 (0.0)	1 (33.3)	0 (0.0)	5 (12.5)	1 (2.6)	12 (50.0)	0 (0.0)	0 (0.0)	0 (0.0)	0 (0.0)
*seb*	3 (1.7)	0 (0.0)	0 (0.0)	0 (0.0)	0 (0.0)	0 (0.0)	2 (5.3)	0 (0.0)	0 (0.0)	1 (16.7)	0 (0.0)	0 (0.0)
*sec*	13 (7.4)	0 (0.0)	3 (42.9)	0 (0.0)	3 (21.4)	1 (2.5)	4 (10.5)	1 (4.2)	1 (6.7)	0 (0.0)	0 (0.0)	0 (0.0)
*sed*	3 (1.7)	0 (0.0)	0 (0.0)	0 (0.0)	0 (0.0)	1 (2.5)	1 (2.6)	1 (4.2)	0 (0.0)	0 (0.0)	0 (0.0)	0 (0.0)
*seh*	4 (2.3)	0 (0.0)	0 (0.0)	0 (0.0)	1 (7.1)	1 (2.5)	2 (5.3)	0 (0.0)	0 (0.0)	0 (0.0)	0 (0.0)	0 (0.0)
*sej*	3 (1.7)	0 (0.0)	0 (0.0)	0 (0.0)	0 (0.0)	1 (2.5)	1 (2.6)	1 (4.2)	0 (0.0)	0 (0.0)	0 (0.0)	0 (0.0)
*sek*	1 (0.6)	0 (0.0)	0 (0.0)	0 (0.0)	0 (0.0)	0 (0.0)	1 (2.6)	0 (0.0)	0 (0.0)	0 (0.0)	0 (0.0)	0 (0.0)
*sel*	13 (7.4)	0 (0.0)	3 (42.9)	0 (0.0)	2 (14.3)	1 (2.5)	5 (13.2)	1 (4.2)	1 (6.7)	0 (0.0)	0 (0.0)	0 (0.0)
*sep*	30 (17.1)	1 (12.5)	0 (0.0)	0 (0.0)	2 (14.3)	11 (27.5)	4 (10.5)	5 (20.8)	3 (20.0)	1 (16.7)	2 (25.0)	1 (8.3)
*seq*	1 (0.6)	0 (0.0)	0 (0.0)	0 (0.0)	1 (7.1)	0 (0.0)	0 (0.0)	0 (0.0)	0 (0.0)	0 (0.0)	0 (0.0)	0 (0.0)
*ser*	3 (1.7)	0 (0.0)	0 (0.0)	0 (0.0)	0 (0.0)	1 (2.5)	1 (2.6)	1 (4.2)	0 (0.0)	0 (0.0)	0 (0.0)	0 (0.0)

^1^ *n* (%); MA3—medical analytics, 3rd year; MA5—medical analytics, 5th year; MB2—medical biotechnology, 2nd year of first-cycle; D1—dietetics, 1st year; MED2—medicine, 2nd year; MED3—medicine, 3rd year; DENT2—dentistry, 2nd year; NUR1—nursing, 1st year; MID1—midwifery, 1st year; MID2—midwifery, 2nd year; PAR2—paramedic, 2nd year.

**Table 5:** Prevalence of the *egc* gene cluster combinations among *S. aureus* strains according to study.

Study	*egc* Gene Cluster					
	*g i m n o u* *n* = 4 ^1^	*g i m n o* *n* = 41 ^1^	*g i m o u* *n* = 32 ^1^	*g i m o* *n* = 1 ^1^	*g i o u* *n* = 16 ^1^	Non-*egc* *n* = 81 ^1^
MA3	0 (0.0)	3 (37.5)	3 (37.5)	0 (0.0)	0 (0.0)	2 (25.0)
MA5	0 (0.0)	5 (71.4)	0 (0.0)	0 (0.0)	1 (14.3)	1 (14.3)
MB2	0 (0.0)	0 (0.0)	2 (66.7)	0 (0.0)	0 (0.0)	1 (33.3)
D1	0 (0.0)	4 (28.6)	4 (28.6)	0 (0.0)	0 (0.0)	6 (42.9)
MED2	0 (0.0)	6 (15.0)	0 (0.0)	1 (2.5)	10 (25.0)	23 (57.5)
MED3	2 (5.3)	10 (26.3)	13 (34.2)	0 (0.0)	0 (0.0)	13 (34.2)
DENT2	0 (0.0)	4 (16.7)	7 (29.2)	0 (0.0)	0 (0.0)	13 (54.2)
NUR1	1 (6.7)	4 (26.7)	0 (0.0)	0 (0.0)	1 (6.7)	9 (60.0)
MID1	1 (16.7)	2 (33.3)	0 (0.0)	0 (0.0)	1 (16.7)	2 (33.3)
MID2	0 (0.0)	1 (12.5)	0 (0.0)	0 (0.0)	3 (37.5)	4 (50.0)
PAR2	0 (0.0)	2 (16.7)	3 (25.0)	0 (0.0)	0 (0.0)	7 (58.3)

^1^ *n* (%); MA3—medical analytics, 3rd year; MA5—medical analytics, 5th year; MB2—medical biotechnology, 2nd year of first-cycle; D1—dietetics, 1st year; MED2—medicine, 2nd year; MED3—medicine, 3rd year; DENT2—dentistry, 2nd year; NUR1—nursing, 1st year; MID1—midwifery, 1st year; MID2—midwifery, 2nd year; PAR2—paramedic, 2nd year.

**Table 6:** Prevalence of *agr* system genes among *S. aureus* strains according to study.

Study	*agr* System Genes			
	*agr-1* *n* = 87 ^1^	*agr-2* *n* = 35 ^1^	*agr-3* *n* = 50 ^1^	*agr-4* *n* = 3 ^1^
MA3	4 (50.0)	1 (12.5)	3 (37.5)	0 (0.0)
MA5	6 (85.7)	0 (0.0)	1 (14.3)	0 (0.0)
MB2	1 (33.3)	0 (0.0)	2 (66.7)	0 (0.0)
D1	7 (50.0)	2 (14.3)	5 (35.7)	0 (0.0)
MED2	21 (52.5)	8 (20.0)	11 (27.5)	0 (0.0)
MED3	16 (42.1)	8 (21.1)	13 (34.2)	1 (2.6)
DENT2	13 (54.2)	4 (16.7)	7 (29.2)	0 (0.0)
NUR1	7 (46.7)	6 (40.0)	1 (6.7)	1 (6.7)
MID1	2 (33.3)	2 (33.3)	1 (16.7)	1 (16.7)
MID2	3 (37.5)	2 (25.0)	3 (37.5)	0 (0.0)
PAR2	7 (58.3)	2 (16.7)	3 (25.0)	0 (0.0)

^1^ *n* (%); MA3—medical analytics, 3rd year; MA5—medical analytics, 5th year; MB2—medical biotechnology, 2nd year of first-cycle; D1—dietetics, 1st year; MED2—medicine, 2nd year; MED3—medicine, 3rd year; DENT2—dentistry, 2nd year; NUR1—nursing, 1st year; MID1—midwifery, 1st year; MID2—midwifery, 2nd year; PAR2—paramedic, 2nd year.

## Data Availability

The datasets generated during the current study are provided in the Supplementary Materials and are also publicly available in the Zenodo repository under the https://doi.org/10.5281/zenodo.18104961.

## Supplementary Materials

The following supporting information can be downloaded at: https://www.mdpi.com/article/10.3390/toxins18050237/s1, Table S1. Primer sequences, product sizes, and control strains used in Single PCR assays [57,85,86]. Table S2. Primer sequences, product sizes, and control strains used in Multiplex PCR assays [87]. Table S3. Volumes of reagents used in the Multiplex PCR reactions. Table S4. Distribution of virulence genes and resistance phenotypes among *S. aureus* strains with different restriction profiles, unique strains (Un), and non-typeable strains (NT).

## Abbreviations

The following abbreviations are used in this manuscript:

AST	Antimicrobial susceptibility testing
CC	Clindamycin
CIP	Ciprofloxacin
cMLS_B_	Constitutive macrolide-lincosamide-streptogramin B resistance phenotype
iMLS_B_	Inducible macrolide-lincosamide-streptogramin B resistance phenotype
MLS_B_	Macrolide-lincosamide-streptogramin B
MS_B_	Macrolide-streptogramin B resistance phenotype
DNA	Deoxyribonucleic acid
E	Erythromycin
ETs	Exfoliative toxins
FOX	Cefoxitin
MRSA	Methicillin-resistant *Staphylococcus aureus*
MSSA	Methicillin-susceptible *Staphylococcus aureus*
MUP	Mupirocin
NT	Non-typeable
PCR	Polymerase chain reaction
PFGE	Pulsed-field gel electrophoresis
PVL	Panton-Valentine leukocidin
R	Resistance
S	Susceptibility
SAgs	Superantigens
Sab	Similarity coefficient
SEs	Staphylococcal enterotoxins
SXT	Trimethoprim/sulfamethoxazole
TSST-1	Toxic shock syndrome toxin-1
