# Supplementary material for: Phenotypic and Genotypic Characteristics of Staphylococcus aureus Nasal Strains Isolated from Students of the Pomeranian Medical University in Szczecin, Poland: A Cross-Sectional Study
Source: Toxins (Basel). 2026 May 21;18(5):237. doi: 10.3390/toxins18050237 (PMC13211644; doi:10.3390/toxins18050237)
Supplement: Supplementary file 1 [file toxins-18-00237-s001.zip › toxins-4286372-supplementary.pdf]

Supplementary Materials: Phenotypic and Genotypic Characteristics of *Staphylococcus aureus* Nasal Strains Isolated From Students of the Pomeranian Medical University in Szczecin, Poland: A Cross-sectional Study

Paweł Kwiatkowski, Helena Masiuk, Agata Pruss, Stefania Giedrys-Kalemba, Piotr Baszuk, Iwona Wojciechowska-Koszko and Monika Sienkiewicz

Table S1. Primer sequences, product sizes, and control strains used in Single PCR assays.

Gene	Primer	Sequence (5'–3')	Product size (bp)	Control strain	Reference
nuc	nuc1	GCG ATT GAT GGT GAT ACG GTT	279	*S. aureus* ATCC 29213	[85]
	nuc2	AGC CAA GCC TTG ACG AAC TAA AGC			
lukS-PV/lukF-PV	pvl1	ATC ATT AGG TAA AAT GTC TGG ACA TGA TCC A	433	*S. aureus* ATCC 25923	[86]
	pvl2	GCA TCA AAT GTA TTG GAT AGC AAA AGC			
hla	hla1	CTG ATT ACT ATC CAA GAA ATT CGA TTG	209	*S. aureus* FRI 913	[57]
	hla2	CTT TCC AGC CTA CTT TTT TAT CAG T			
hlb	hlb1	GTG CAC TTA CTG ACA ATA GTG C	309	*S. aureus* NCTC 7428	
	hlb2	GTT GAT GAG TAG CTA CCT TCA GT			
hld	hld1	AAG AAT TTT TAT CTT AAT TAA GGA AGG AGT G	111	*S. aureus* NCTC 9393	
	hld2	TTA GTG AAT TTG TTC ACT GTG TCG A			
hlg	hlg1	GTC ATA GAG TCC ATA ATG CAT TTA A	535	*S. aureus* ATCC 49775	
	hlg2	CAC CAA ATG TAT AGC CTA AAG TG			
etb	etb1	CAG ATA AAG AGC TTT ATA CAC ACA TTA C	612	*S. aureus* TC146	
	etb2	AGT GAA CTT ATC TTT CTA TTG AAA AAC ACT C			

Legend: A – adenine, T – thymine, G – guanine, C – cytosine, bp – base pairs

Table S2. Primer sequences, product sizes, and control strains used in Multiplex PCR assays.

Multiplex	Gene	Primer	Sequence (5'–3')	Product size (bp)	Control strain	Reference
I	*sea*	sea1	GAA AAA AGT CTG AAT TGC AGG GAA CA	560	*S. aureus* FRI913	[87]
		sea2	CAA ATA AAT CGT AAT TAA CCG AAG GTT C			
	seh	seh1	CAA TCA CAT CAT ATG CGA AAG CAG	376	*S. aureus* FRI137	
		seh2	CAT CTA CCC AAA CAT TAG CAC C			
	sec	sec1	CTT GTA TGT ATG GAG GAA TAA CAA AAC ATG	275	*S. aureus* FRI913	
		sec2	CAT ATC ATA CCA AAA AGT ATT GCC GT			
	tst	tst1	TTC ACT ATT TGT AAA AGT GTC AGA CCC ACT	180		
		tst2	TAC TAA TGA ATT TTT TTA TCG TAA GCC CTT			
II	*sed*	sed1	GAA TTA AGT AGT ACC GCG CTA AAT AAT ATG	492	*S. aureus* FRI1151m	
		sed2	GCT GTA TTT TTC CTC CGA GAG T			
	etd	etd1	CAA ACT ATC ATG TAT CAA GGA TGG	358	*S. aureus* TY114	
		etd2	CCA GAA TTT CCC GAC TCA G			
	eta	eta1	ACT GTA GGA GCT AGT GCA TTT GT	190	*S. aureus* A920210	
		eta2	TGG ATA CTT TTG TCT ATC TTT TTC ATC AAC			
	sek	sek1	ATG CCA GCG CTC AAG GC	134	*S. aureus* FRI913	
		sek2	AGA TTC ATT TGA AAA TTG TAG TTG ATT AGC T			
		sek3	TGC CAG CGC TCA AGG TG			
III	*see*	see1	CAA AGA AAT GCT TTA AGC AAT CTT AGG C	482		
		see2	CAC CTT ACC GCC AAAGCT G			
	seb	seb1	ATT CTA TTA AGG ACA CTA AGT TAG GGA	404	*S. aureus* CCM5757	
		seb2	ATC CCG TTT CAT AAG GCG AGT			
	sem	sem1	CTA TTA ATC TTT GGG TTA ATG GAG AAC	326	*S. aureus* FRI137	
		sem2	TTC AGT TTC GAC AGT TTT GTT GTC AT			
	sel	sel1	GCG ATG TAG GTC CAG GAA AC	234		
		sel2	CAT ATA TAG TAC GAG AGT TAG AAC CAT A			
	seo	seo1	AGT TTG TGT AAG AAG TCA AGT GTA GA	180		
		seo2	ATC TTT AAA TTC AGC AGA TAT TCC ATC TAA C			
IV	*sen*	sen1	CGT GGC AAT TAG ACG AGT C	474		
		sen2	GAT TGA TCT TGA TGA TTA TGA G			
	seg	seg1	TCT CCA CCT GTT GAA GG	323		
		seg2	AAG TGA TTG TCT ATT GTC G			
	seq	seq1	ACC TGA AAA GCT TCA AGG A	204	*S. aureus* Col	
		seq2	CGC CAA CGT AAT TCC AC			

	sej	sej1	TCA GAA CTG TTG TTC CGC TAG	138	*S. aureus* FRI1151m	
		sej2	GAA TTT TAC CAT CAA AGG TAC			
V	*sei*	sei1	CTC GAA TTT TCA ACA GGT AC	461	*S. aureus* FRI137	
		sei2	AGG CAG TCC ATC TCC TG			
	ser	ser1	AGC GGT AAT AGC AGA AAA TG	363	*S. aureus* FRI1151 m	
		ser2	TCT TGT ACC GTA ACC GTT TT			
	seu	seu1	AAT GGC TCT AAA ATT GAT GG	215	*S. aureus* FRI137	
		seu2	ATT TGA TTT CCA TCA TGC TC			
	sep	sep1	GAA TTG CAG GGA ACT GCT	182	*S. aureus* N315	
		sep2	GGC GGT GTC TTT TGA AC			
VI	*agr 1-4*	pan agr	ATG CAC ATG GTG CAC ATG C			
	agr-1	agr1	GTC ACA AGT ACT ATA AGC TGC GAT	439	*S. aureus* Col	
	agr-2	agr2	TAT TAC TAA TTG AAA AGT GCC ATA GC	572	*S. aureus* N315	
	agr-3	agr3	GTA ATG TAA TAG CTT GTA TAA TAA TAC CCA G	320	*S. aureus* TY114	
	agr-4	agr4	CGA TAA TGC CGT AAT ACC CG	657	*S. aureus* A920210	

Legend: A – adenine, T – thymine, G – guanine, C – cytosine, bp – base pairs

Table S3. Volumes of reagents used in the Multiplex PCR reactions.

Multiplex	Reaction buffer (µl)	dNTPs (µl)	MgCMED2 (µl)	Primers		GoTaq polymerase (µl)	H_2O (µl)
				Primer name	Primer volume (µl)		
I	5.0	0.25	1.0	sea1/2, seh1/2, sec1/2, tst1/2	0.75	0.2	11.55
II	5.0	0.25	1.0	sed1/2, etd1/2, eta1/2	0.75	0.2	9.75
				sek1/2/3	1.1		
III	5.0	0.25	1.0	see1/2, seb1/2	0.75	0.2	7.69
				sem1/2, sel1/2	1.0		
				seo1/2	1.43		
IV	5.0	0.25	1.0	seg1/2, seq1/2	0.75	0.2	8.55
				sej1/2	1.0		
				sen1/2	2.0		
V	5.0	0.25	1.0	seu1/2, ser1/2, sep1/2	0.75	0.2	11.05
				sei1/2	1.0		
VI	5.0	0.25	1.0	pan agr, agr1/2/3/4	1.5	0.2	10.05

Table S4. Distribution of virulence genes and resistance phenotypes among *S. aureus* strains with different restriction profiles, unique strains (Un), and non-typeable strains (NT).

Genotype	Subtype	Number of strains *n* (%)	Field and year of study	*agr*	Hemolysins	SEs		*ETs*	*tst*	*lukS-PV/lukF-PV*	Antibiotic resistance (phenotype)
						egc	other				
A	A1	2 (1.1)	MED2	1	*a d g*	*g i m n o*	-	-	-	-	E, CC (iMLS$_B$)
	A2		D1	1	*a d g*	*g i m n o*	*c l*	-	-	-	-
B	B1	2 (1.1)	MED2	2	*a d g*	-	-	-	-	-	-
	B2			1	*a d g*	-	*a*	-	-	-	-
C	C1	2 (1.1)	MED2	2	*a d g*	-	-	-	-	-	-
	C2			2	*a d g*	-	-	-	-	-	-
D	D1	2 (1.1)	MID1	2	*a d*	*g i m n o*	-	-	-	-	SXT
	D2		MED3	3	*a d g*	*g i m o u*	*h k*	-	+	-	-
E	E1	3 (1.7)	NUR1	1	*a d g*	*g i m n o*	*c l*	-	-	-	-
	E2			1	*a d g*	*g i m n o*	-	-	-	-	-
	E3		MED2	1	*a d g*	*g i m n o*	*c l*	-	-	-	-
F	F1	2 (1.1)	MED2	1	*a d*	-	-	-	-	-	-

	F2			3	*a d*	-	*h*	-	-	-	-
G	G1	3 (1.7)	MED2	1	*a d g*	*g i m o*	-	-	-	-	-
	G2		MED3	1	*a d g*	*g i m n o*	*b*	*etd*	-	-	-
	G3			2	*a d g*	-	*l p*	-	-	-	-
H	H1	2 (1.1)	MED3	1	*a d g*	-	-	-	-	-	-
	H2		DENT2	1	*a b d g*	-	-	-	-	-	-
I	I1	2 (1.1)	MED3	1	*a d g*	-	-	-	-	-	-
	I2		MID1	2	*a d g*	-	*b p*	-	-	-	E, CC (iMLS$_B$)
J	J1	2 (1.1)	PAR2	1	*a d g*	-	-	-	-	-	E, CC (iMLS$_B$)
	J2			1	*a d g*	-	-	-	-	-	-
K	K1	2 (1.1)	MED3	3	*a d g*	*g i m o u*	-	-	-	-	-
	K2		DENT2	1	*a d g*	-	*a p*	-	-	-	E, CC (iMLS$_B$)
L	L1	2 (1.1)	MA3	3	*a d g*	*g i m o u*	-	-	+	-	-
	L2			3	*a d g*	*g i m o u*	-	-	+	-	-
M	M1	3 (1.7)	DENT2	2	*a d g*	-	-	-	-	-	-
	M2			2	*a d g*	-	-	-	-	-	-
	M3		MA3	2	*a d g*	-	-	-	-	-	-
N	N1	9 (5.1)	MED2	1	*a d g*	-	*p*	-	-	-	-
	N2			1	*a d g*	-	*p*	-	-	-	-
	N3			1	*a d g*	-	-	-	-	-	-
	N4			1	*a d g*	-	*p*	-	-	-	-
	N6			1	*a d g*	-	*p*	-	-	-	E, CC (iMLS$_B$)
	N7			1	*a d g*	-	*p*	-	-	-	-
	N8			2	*a d g*	*g i m n o*	-	-	-	-	-
	N9			2	*a d g*	*g i m n o*	*d j r*	-	-	-	E, CC (iMLS$_B$)
	N5		NUR1	1	*a d g*	-	-	-	-	-	-
O	O1	2 (1.1)	MED2	1	*a d*	-	-	-	-	-	-
	O2			1	*a d g*	*g i m n o*	-	*etd*	-	-	-
P	P1	2 (1.1)	MED2	1	*a d g*	-	*p*	-	-	-	-
	P2			1	*a d g*	-	*p*	-	-	-	-
Q	Q1	2 (1.1)	NUR1	1	*a d*	-	-	-	-	-	-
	Q2		PAR2	1	*a g*	-	-	-	-	-	-
R	R1	3 (1.7)	NUR1	1	*a d g*	-	*p*	-	-	-	-
	R2		DENT2	1	*a d g*	-	*p*	-	-	-	-
	R3		MED3	1	*a d g*	-	*p*	-	-	-	-

S	S1	3 (1.7)	DENT2	1	*a d g*	-	*a p*	-	-	-	-
	S2		MB2	3	*a d g*	*g i m o u*	-	-	+	-	-
	S3		MID2	2	*a d g*	-	-	-	-	-	-
T	T1	3 (1.7)	PAR2	2	*a g*	-	-	-	-	-	E, CC (iMLS$_B$)
	T2			1	*a g*	-	*p*	-	-	-	-
	T3		MED3	1	*a d g*	-	*p*	-	-	-	-
U	U1	3 (1.7)	MED3	1	*a d g*	-	-	-	-	-	-
	U2			1	*a d g*	-	-	-	-	-	-
	U3		MED2	1	*a d g*	-	*p*	-	-	-	-
V	V1	5 (2.9)	MID2	1	*a d g*	-	*p*	-	-	-	-
	V2			1	*a d g*	-	*p*	-	-	-	-
	V3		DENT2	1	*a d g*	-	*a p*	-	-	-	-
	V5			1	*a d g*	-	*a p*	-	-	-	-
	V4		MA3	1	*a d g*	-	*a p*	-	-	-	-
W	W1	5 (2.9)	MED2	1	*a d g*	-	*p*	-	-	-	E, CC (iMLS$_B$)
	W2			1	*a d g*	-	*p*	-	-	-	-
	W5			1	*a d g*	-	*p*	-	-	-	-
	W3		D1	1	*a d g*	-	*p*	-	-	-	-
	W4			1	*a d g*	-	*p*	-	-	-	-
X	X1	2 (1.1)	MED3	2	*a d*	*g i m n o*	*d j r*	-	+	-	-
	X2			2	*a d*	*g i m n o*	-	-	-	-	MUP, E, CC (iMLS$_B$)
Y	Y1	2 (1.1)	NUR1	2	*a d g*	-	-	-	-	-	-
	Y2			2	*a d g*	-	*p*	-	-	-	-
Z	Z1	5 (2.9)	D1	2	*a d g*	-	-	-	-	-	-
	Z2			2	*a d g*	-	-	-	-	-	-
	Z3		MED2	2	*a d g*	-	-	-	-	-	-
	Z5			2	*a d g*	-	-	-	-	-	-
	Z4		MID2	2	*a d g*	-	-	-	-	-	-
AB	AB1	3 (1.7)	MED2	2	*a d g*	-	-	-	-	-	-
	AB2		NUR1	2	*a d g*	-	-	-	-	-	-
	AB3			2	*a d*	-	-	-	-	-	-
AC	AC1	4 (2.3)	MED3	1	*a d g*	*g i m n o*	-	-	-	-	-
	AC2		MA5	1	*a d g*	*g i m n o*	-	-	-	-	-
	AC3		MID1	1	*a d g*	*g i m n o*	-	-	-	-	E, CC (cMLS$_B$)
	AC4		MED2	1	*a d g*	*g i m n o*	-	-	-	-	E, CC (cMLS$_B$)

AD	-	2 (1.1)	MA5	1	*a d g*	*g i m n o*	*c l*	-	-	-	-
				1	*a d g*	*g i m n o*	*c l*	-	-	-	-
AE	AE1	2 (1.1)	MID2	3	*a d g*	*g i o u*	-	-	+	-	-
	AE2		MED3	4	*a d g*	*g i m n o u*	*b*	-	-	-	-
AF	AF1	2 (1.1)	MA3	3	*a d g*	*g i m o u*	*a*	-	-	-	-
	AF2			1	*a d g*	*g i m n o*	*a*	-	-	-	-
AG	AG1	2 (1.1)	DENT2	1	*a b d g*	-	-	-	+	-	-
	AG2			1	*a d g*	-	-	-	-	-	-
AH	AH1	10 (5.7)	MID2	3	*a d g*	*g i o u*	-	-	+	-	-
	AH3			3	*a d g*	*g i o u*	-	-	+	-	-
	AH2		MED3	3	*a d g*	*g i m o u*	-	-	+	-	-
	AH9			3	*a d g*	*g i m o u*	*a*	-	+	-	MUP
	AH10			3	*a d g*	*g i m o u*	-	-	+	-	-
	AH4		DENT2	3	*a d g*	*g i m o u*	*a*	-	+	-	-
	AH5			3	*a d g*	*g i m o u*	*a*	-	+	-	-
	AH6		MID1	3	*a d g*	*g i o u*	-	-	-	-	-
	AH7		MED2	3	*a d g*	*g i o u*	-	-	-	-	-
	AH8			3	*a d g*	*g i o u*	-	-	+	-	-
AI	AI1	2 (1.1)	D1	3	*a d g*	*g i m o u*	-	-	+	-	-
	AI2		MED3	3	*a d g*	*g i m o u*	-	-	-	-	E, CC ($iMLS_B$)
AJ	AJ1	2 (1.1)	MED3	3	*a g*	*g i m o u*	*h*	-	+	-	-
	AJ2		PAR2	3	*a g*	*g i m o u*	-	-	-	-	-
AK	AK1	2 (1.1)	MED3	3	*a d g*	*g i m o u*	-	-	-	-	-
	AK2			3	*a d g*	*g i m o u*	-	-	+	-	MUP
AL	AL1	2 (1.1)	MED2	3	*a d g*	*g i o u*	*a*	-	-	-	-
	AL2			3	*a d g*	*g i o u*	-	-	+	-	-
AM	AM1	4 (2.3)	MED2	3	*a d g*	*g i o u*	-	-	+	-	-
	AM2			3	*a d g*	*g i o u*	*a*	-	-	-	E, CC ($iMLS_B$)
	MA3			3	*a d g*	*g i o u*	-	-	+	-	-
	AM4		NUR1	3	*a d g*	*g i o u*	-	-	+	-	-
AN	AN1	4 (2.3)	DENT2	3	*a d g*	*g i m o u*	-	-	+	-	-
	AN3			3	*a d g*	*g i m o u*	*a*	-	-	-	E, CC ($iMLS_B$)
	AN2		MB2	3	*a d g*	*g i m o u*	-	-	+	-	E, CC ($iMLS_B$)
	AN4		MED2	3	*a d g*	*g i o u*	-	-	+	-	-
AO	AO1	3 (1.7)	PAR2	3	*a b d g*	*g i m o u*	-	-	+	-	-

	AO2			3	*a b d g*	*g i m o u*	-	-	-	-	-
	AO3		MED3	3	*a b d g*	*g i m o u*	-	-	+	-	-
AP	AP1	3 (1.7)	D1	3	*a d g*	*g i m o u*	-	-	-	-	-
	AP2		MED3	3	*a d g*	*g i m o u*	-	-	+	-	E, CC (iMLS$_B$)
	AP3			3	*a d g*	*g i m o u*	-	-	-	-	-
AQ	AQ1	2 (1.1)	NUR1	1	*a d g*	*g i m n o*	-	-	-	-	-
	AQ2		MID2	1	*a d g*	*g i m n o*	-	-	-	-	-
AR	AR1	2 (1.1)	DENT2	3	*a d g*	*g i m o u*	*a*	-	+	-	-
	AR2			3	*a d g*	*g i m o u*	*a*	-	+	-	-
AS	AS1	3 (1.7)	MED3	1	*a d g*	-	-	-	-	-	-
	AS2			1	*a d g*	-	-	-	-	-	-
	AS3			2	*a d g*	-	-	-	-	-	-
AT	AT1	2 (1.1)	PAR2	1	*a g*	*g i m n o*	-	-	-	-	-
	AT2		MED3	2	*a g*	-	-	-	-	-	-
Un	Un1	38 (21.7)	MID1	4	*a d g*	*g i m n o u*	-	-	-	-	-
	Un2		NUR1	4	*a d*	*g i m n o u*	-	*eta*	-	-	-
	Un3		MED3	2	*a g*	*g i m n o u*	-	-	-	-	-
	Un4		DENT2	1	*a d g*	*g i m n o*	*c d j l r*	-	-	-	-
	Un5		D1	1	*a d g*	*g i m n o*	-	-	-	-	-
	Un6		D1	1	*a d g*	*g i m n o*	*c*	-	-	-	-
	Un7		MED2	1	*a d g*	-	-	-	-	-	-
	Un8		D1	3	*a d g*	*g i m o u*	-	-	+	-	-
	Un9		MED2	3	*a d g*	*g i o u*	*a*	-	-	-	E, CC (iMLS$_B$)
	Un10		DENT2	1	*a d g*	*g i m n o*	*a*	-	-	-	-
	Un11		MED3	1	*a d g*	*g i m n o*	*cl*	-	+	-	-
	Un12		MA5	3	*a b d g*	*g i o u*	-	-	+	-	-
	Un13		MED3	2	*a b d*	*g i m n o*	-	-	+	-	-
	Un14		MA5	1	*a d g*	-	-	-	-	-	-
	Un15		NUR1	1	*a d g*	-	*p*	-	-	-	E, CC (iMLS$_B$)
	Un16			2	*a d*	*g i m n o*	-	-	-	-	-
	Un17		D1	1	*a d g*	*g i m n o*	-	*etd*	-	-	-
	Un18			1	*a d g*	-	*c l*	-	+	-	-
	Un19		PAR2	1	*a g*	-	-	-	+	-	-
	Un20			2	*a g*	-	-	-	-	+	-
	Un21		MED3	2	*a d g*	-	-	-	-	-	-

	Un22		DENT2	2	*a d g*	-	-	-	-	-	-
	Un23		MA5	1	*a d g*	*g i m n o*	-	-	-	-	E, CC (iMLS$_B$)
	Un24		MA3	1	*a d g*	*g i m n o*	*a*	-	-	-	-
	Un25			1	*a d g*	*g i m n o*	*a*	-	-	-	-
	Un26		NUR1	2	*a d g*	-	-	-	-	-	-
	Un27		MED2	3	*a d*	*g i o u*	*a*	-	-	-	E, CC (iMLS$_B$)
	Un28		DENT2	3	*a d g*	*g i m o u*	*a*	-	-	-	-
	Un29		MED3	3	*a g*	*g i m o u*	*p*	-	+	-	-
	Un30			1	*a d g*	*g i m n o*	-	-	+	-	-
	Un31		DENT2	2	*a d g*	*g i m n o*	-	*eta*	-	-	E, CC (iMLS$_B$)
	Un32			1	*a d g*	*g i m n o*	-	-	-	-	-
	Un33		MED3	1	*a d g*	*g i m n o*	*c l*	-	-	-	E, CC (iMLS$_B$)
	Un34		D1	3	*a d g*	-	*h q*	-	-	-	-
	Un35		MED3	1	*a d g*	-	-	-	-	-	-
	Un36		MA5	1	*a d g*	*g i m n o*	*c l*	-	-	-	-
	Un37		MED3	1	*a d g*	*g i m n o*	*c l*	-	-	-	-
	Un38		PAR2	1	*a d g*	*g i m n o*	-	-	-	-	-
NT	NT1	6 (3.4)	MB2	1	*a d g*	-	*a*	-	-	-	E, CC (iMLS$_B$)
	NT2		D1	3	*a d g*	*g i m o u*	-	-	+	-	-
	NT3		MED3	1	*a d g*	*g i m n o*	*c l*	-	-	-	-
	NT4		DENT2	1	*a b d g*	-	-	-	-	-	CIP
	NT5			1	*a b d g*	-	*a*	-	-	-	-
	NT6		MID1	1	*a d g*	-	-	-	-	-	E, CC (iMLS$_B$)

Legend: MA3 - medical analytics, 3rd year; MA5 - medical analytics, 5th year; MB2 - medical biotechnology, 2nd year of first-cycle; D1 - dietetics, 1st year; MED2 - medicine, 2nd year; MED3 - medicine, 3rd year; DENT2 - dentistry, 2nd year; NUR1 - nursing, 1st year; MID1 - midwifery, 1st year; MID2 - midwifery, 2nd year; PAR2 - paramedic, 2nd year; *agr* - accessory gene regulator system of virulence gene expression; *a-d* - genes encoding hemolysins; *egc* - enterotoxin gene cluster; *eta-etd* - genes encoding ETs (exfoliative toxins); SEs - staphylococcal enterotoxins; *tst* - gene encoding toxic shock syndrome toxin; *lukS-PV/lukF-PV* - gene encoding Panton-Valentine leukocidin; E - erythromycin, CC - clindamycin, CIP - ciprofloxacin, MUP - mupirocin, SXT - trimethoprim/sulfamethoxazole, iMLS$_B$ - inducible macrolide-lincosamide-streptogramin B (MLS$_B$) resistance phenotype, cMLS$_B$ - constitutive macrolide-lincosamide-streptogramin B (MLS$_B$) resistance phenotype; Un - unique strain; NT - non-typeable strain.
